# Ultrasound Pulse Emission Spectroscopy Method to Characterize Xylem Conduits in Plant Stems

**DOI:** 10.34133/2022/9790438

**Published:** 2022-09-13

**Authors:** Satadal Dutta, Zhiyi Chen, Elias Kaiser, Priscilla Malcolm Matamoros, Peter G. Steeneken, Gerard J. Verbiest

**Affiliations:** ^1^Department of Precision and Microsystems Engineering, Faculty of 3ME, TU Delft, Mekelweg 2, 2628CD Delft, Netherlands; ^2^Horticulture and Product Physiology, Department of Plant Sciences, Wageningen University and Research, Droevendaalsesteeg 1, 6708PB Wageningen, Netherlands

## Abstract

Although it is well known that plants emit acoustic pulses under drought stress, the exact origin of the waveform of these ultrasound pulses has remained elusive. Here, we present evidence for a correlation between the characteristics of the waveform of these pulses and the dimensions of xylem conduits in plants. Using a model that relates the resonant vibrations of a vessel to its dimension and viscoelasticity, we extract the xylem radii from the waveforms of ultrasound pulses and show that these are correlated and in good agreement with optical microscopy. We demonstrate the versatility of the method by applying it to shoots of ten different vascular plant species. In particular, for *Hydrangea quercifolia*, we further extract vessel element lengths with our model and compare them with scanning electron cryomicroscopy. The ultrasonic, noninvasive characterization of internal conduit dimensions enables a breakthrough in speed and accuracy in plant phenotyping and stress detection.

## 1. Introduction

The generation of sound waves by plants has received considerable attention during the last decades [1, 2]. Several studies have investigated the mechanism of sound generation and found that it is strongly related to the mechanism of water transport in plants [3], which is vital to our understanding of plant function and stress resilience [4, 5]. In vascular plants, the xylem is responsible for water and nutrient transport from the roots to the leaves [3]. Evaporation of water from leaves (transpiration) results in a tensile force on the water column, which, combined with the strong cohesion of water molecules, results in ascent of water from the roots to the leaves [6]. During drought or high transpiration rate, the tension in the water column can increase rapidly. Movement of water through these cellular conduits bound by porous and granular media can lead to several kinds of acoustic interactions. Primarily, the tensile stress is released by the formation of vapor or air bubbles [6–8] in the xylem. This bubble formation results in a sudden release of the elastic energy stored in the water column, a fraction of which is converted to a sound pulse [9]. The rate at which such pulses are emitted has been used as an indicator of a plant's response and vulnerability to drought stress [10–13]. The time- and frequency-domain features of these ultrasound pulses were measured directly from (intact) plant shoots [14] and from sapwood specimens [15] in recent studies. In, yet, another study, the amplitude of the ultrasound signals from leaves was linked to the leaf water potential, hydraulic conductance, and conduit diameter [16]. Yet, the physical origin and relevance of the observed waveforms in these acoustic pulses [17–20] has remained elusive.

**Figure 1:**
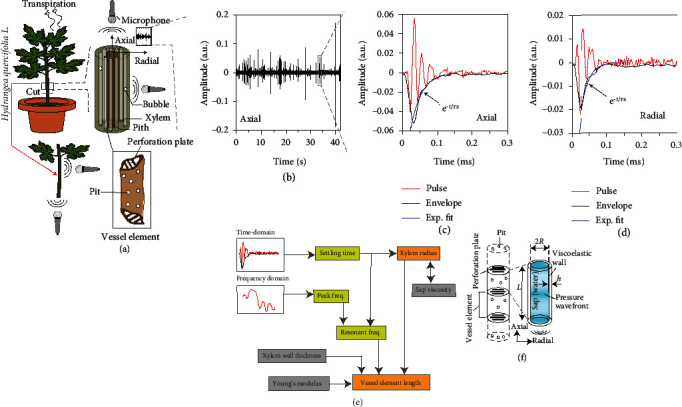
Ultrasound measurement set-up, recorded waveforms, and analysis method. (a) Schematic set-up for recording of ultrasound pulses from shoots of *Hydrangea quercifolia* along axial and radial directions. The zoom-in represents a schematic of the vascular bundle of the stem, showing the peripheral arrangement of tubular xylem vessels around the core. Each xylem vessel is composed of a network of several vessel elements interconnected via perforation plates as illustrated in the box. (b) Example raw time-domain data for ultrasound recorded for a duration of 70 s along the axial direction of a stem. Time *t* = 0 represents the start of the recording, which occurs after ~5 minutes of drying. (c and d) Zoomed-in time-domain example ultrasound pulses recorded axially and radially, respectively. Black curves represent the amplitude envelope that decays exponentially (damping), and blue curves represent the exponential fit of the envelope decay. (e) Schematic flowchart illustrating the steps in our analysis. The settling time and peak frequencies are obtained from the time-domain and frequency-domain waveforms (Figures 2(e)–2(h) and 3(a)–3(d)). The resonant frequency is obtained from peak frequency and settling time (see Materials and Methods). Using these, xylem conduit radius and xylem vessel element length are extracted. Parameters of sap viscosity, vessel wall thickness, and Young's modulus are taken as input. (f) Schematic of water-conducting xylem vessels in vascular plants. They consist of a series network of vessel members/elements, which are interconnected via perforation plates. Also shown is the simplified cylindrical acoustic resonator model for a xylem conduit sustaining damped longitudinal standing waves in its sap.

The xylem is a major player in determining a plant's response to biotic and abiotic stresses, e.g., availability of water, and pathogen infection. Monitoring xylem conduit traits is thus beneficial for plant phenotyping. Current knowledge of the xylem conduit dimensions has mainly been dependent on microscopy techniques. Conduits are of two types: vessels and tracheids. Xylem vessels resemble cylindrical tubes with fused ends [21]. These tubes consist of several xylem vessel elements that are separated by perforation plates (see Figure 1(a)). Diameters of these vessels can range from ~1 *μ*m in small herbs to ~100 *μ*m in shrubs and small trees. Their lengths can range from ~100 *μ*m to ~10 cm [22–24], across herbaceous and shrub-like plants. The viscoelastic walls of xylem vessels are composed of cellulose, hemicellulose, pectin, and lignin, which can have a wide range of elastic moduli depending on their relative composition [25] and the water content [26–28]. The elasticity of macroscopic segments of plant stems can be measured via various mechanical loading techniques [28, 29], which are invasive. Existing techniques to measure xylem dimensions, such as paint injection, X-ray microcomputed tomography (CT), optical microscopy, and scanning electron microscopy [30–33], are also destructive and time-consuming (~30 minutes per sample for CT and ~12–24 hours per sample for microscopy).

Here, we present and experimentally validate a physical model that links xylem vessel dimensions and (visco-)elasticity, to the time and frequency characteristics of measured ultrasound pulses. Using *Hydrangea quercifolia* (H.q.) as our main test species, we compare information about the radius, length, and viscoelasticity of xylem vessels obtained by analysing ultrasound pulses, to that gained by independent destructive techniques, namely, latex paint staining and cross-section optical microscopy, scanning electron microscopy, and uniaxial tensile loading. In addition, we further elucidate the correlation between viscous damping in the ultrasound pulses and the xylem vessel radius distribution by experiments on eight additional angiosperm species, namely, *Artemisia annua* (A.a.), *Begonia maculata* (B.m.), *Plectranthus scutellarioides* (P.s.), *Impatiens hawkeri* (I.h.), *Salvia officinalis* (S.o.), *Cucumis sativus* (C.s.), *Capsicum annuum* (C.a.), and *Solanum lycopersicum* (S.l.). We also included a coniferous shrub *Cephalotaxus harringtonia* (C.h.), in this study to also validate our method on a gymnosperm, which contains tracheids instead of vessel elements.

## 2. Results

Our study consists of two parts: (i) recording and analysing ultrasound pulses from drying plant shoots and (ii) validating the analytical results with destructive characterization on stem segments. Results is organized as follows: firstly, we analyse the time-domain features of the recorded ultrasound pulses. Secondly, we develop an analytical model to extract xylem vessel radius from the settling time of these ultrasound pulses. The obtained xylem vessel radii are validated by optical microscopy. Thirdly, we study the resonance frequencies present in the pulses and show the correlation with pulse settling time and thereby the acoustic vessel radius. In particular, we illustrate, using Hydrangea as an example species, that we can extract the length of vessel elements using our model, in combination with measured elasticity of stem segments. Lastly, we merge our findings to create an experimental map of acoustic lengths and radii of xylem vessels in Hydrangea.

### 2.1. Recording Ultrasound Waveforms

We first examined the waveforms of ultrasound pulses emitted by cut plant shoots. A total of three specimens, samples A, B, and C, were taken from three Hydrangea plants (see Materials and Methods). Ultrasound pulses were recorded with a broadband ultrasound microphone placed along the axial and radial direction of the stem, as shown in Figure 1(a). While the axial recording generally helps in detecting louder sound bursts (larger amplitudes), the radial recording is relevant from the viewpoint of noninvasive detection. The microphone recorded the time series of the ultrasound emissions starting ~5 minutes into the drying process (see Figure 1(b) and Materials and Methods), where time *t* = 0 s corresponds to the start of the recording. The pulses occur sporadically and with varying amplitudes. We observe that the time-domain waveforms of these pulses resemble damped oscillations, both when recorded along the axial and in the radial directions (Figures 1(c) and 1(d)). The pulse amplitude in time-domain decays exponentially with time constant *τ*_s_ (settling time). The *τ*_s_ for the many individually measured axial and radial sound pulses of all the three stem samples A, B, and C are shown in Figures S1. Values of settling times recorded in axial and radial directions are observed to be statistically similar. The determined settling times of samples B and C agree with those of sample A, showing statistical similarity. All pulses die out within ~0.5 ms, as found in isolated conifer sapwood, in an earlier work [9]. Based on this observation, we hypothesize that the damped oscillations are generated by resonant vibrations within the xylem vessels. In the following paragraphs, we develop a micromechanical model of the xylem vessel element. In order to validate the model, we extract the settling times and characteristic frequencies of ultrasound pulse waveforms. These are subsequently interpreted to estimate xylem vessel dimensions (see Figure 1(e)).

### 2.2. Model for Pulse Settling Time and Ultrasound Frequency versus Xylem Vessel Radius

In order to explain the origin of the observed ultrasound waveforms and to use them to extract information about the plant's microstructure, we develop a model relating the micromechanics of the xylem to the waveform of the generated ultrasound. We hypothesize that the damped oscillations are identical to those of an organ pipe filled with water [34]. The bubble formation excites axial standing waves in the sap (water), whose resonance frequencies depend on the longitudinal speed of sound in the pipe *v*_eff_ and the xylem vessel element length *L* (see Materials and Methods). We model the xylem vessel as a resonant cylindrical pipe containing a series network of vessel elements of length *L*, which are bounded by perforation plates [3, 30] (see Figure 1(f)). The perforation plates serve as nonideal (leaky) reflecting surfaces at the termination of a vessel element for the pressure waves. The sound waves propagate along the length of the xylem vessel and are likely to dominate the recorded ultrasound. These waves undergo damping, primarily due to the dynamic viscosity of sap (water) *η*_l_ in the xylem, which dominates the settling time *τ*_s_. The resonating element is described using a linear second-order resonator model consisting of lumped acoustic inductance, capacitance, and resistance (see Materials and Methods). Using this acoustic model, we express the effective xylem radius *R* as (4*η*_l_*τ*_s_/*ρ*_l_)^0.5^, where *ρ*_l_ is the mass density of sap (water) in the xylem (see equation (8) in Materials and Methods). In this model, *R* is calculated independently of length *L*, from the settling time of the measured time-domain waveform.

**Figure 2:**
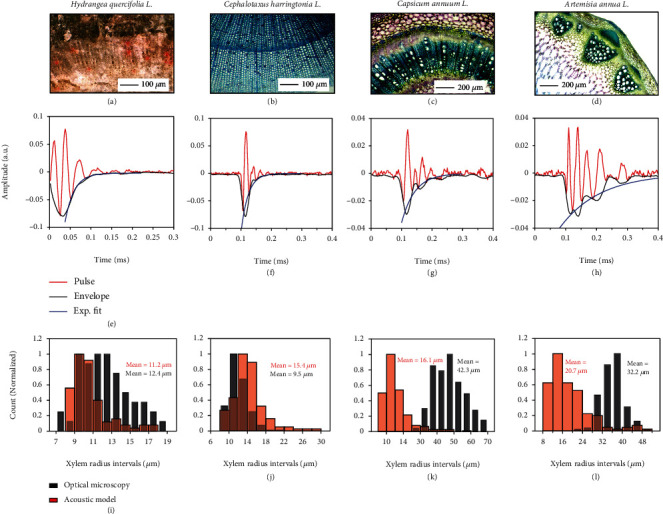
Xylem vessel radius extraction from damping in axial sound waves. (a–d) Optical micrographs of the transverse section of stem samples of the indicated species, showing the stained xylem vessels (with latex paint in (a) and with toluidine blue in (b)–(d)). (e–h) Example time-domain waveforms (in red) of recorded pulses from each of the indicated plant species. The black curves represent the pulse envelope, and the blue curve represents the exponential fit on the decaying envelope. (i–l) Histogram showing the model-extracted xylem radii (in red) and that of the observed xylem radii (in black) obtained via optical microscopy for the indicated species.

To validate our model, we obtained micrographs of transverse cross-sections of the stem samples via optical microscopy (Figures 2(a)–2(d) and Figure S2). Ultrasound waveforms were recorded from a total of three shoot samples from each plant species. Example time-domain waveforms are shown in Figures 2(e)–2(h). Next, the histograms of xylem radii *R* are extracted using the acoustic model from the axially recorded ultrasound pulses (by combining data from the three shoot samples) and also from the optical micrographs. As shown in Figure 2(i), the mean (± standard error (s.e.)) acoustic *R* for Hydrangea is found to be 11.2 ± 0.5 *μ*m. The observed vessel radius *R* via optical microscopy is 12.4 ± 0.3 *μ*m. Similar data are obtained for the remaining species (Figures 2(j)–2(l), Figures S3-S11, and Table 1). The relatively longer *τ*_s_ for Capsicum and Artemisia are in agreement with its wider mean vessel radii, compared to that of Hydrangea and the gymnosperm Cephalotaxus (Table 1).

**Figure 3:**
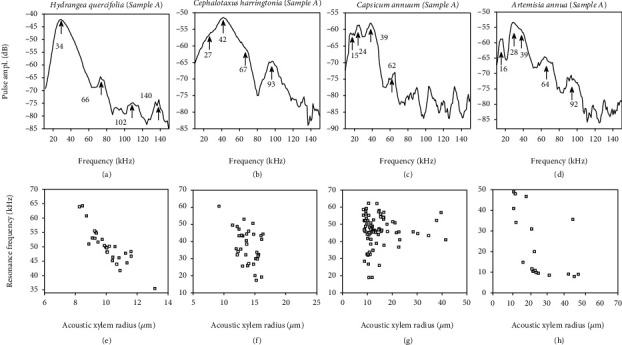
Ultrasound frequency spectra, resonance frequency, and acoustic xylem radius. (a–d) Observed characteristic peak frequencies (arrowheads) in the example Fourier transform of the ultrasound pulses recorded axially from the indicated plant species. (e–h) Model-extracted resonance frequency versus acoustic xylem radius for sound pulses from stem sample A of each of the indicated plant species. Resonance frequency is obtained from the peak frequency of highest amplitude in the recorded pulses.

Fourier spectra of the representative ultrasound pulses (recorded axially) exhibit characteristic peak frequencies (Figures 3(a)–3(d)). For example, the peak frequency with the largest amplitude (*f*_p(axial)_) in the pulse from sample A of Hydrangea occurs at 34 kHz. The same occurs at 42 kHz, 39 kHz, and 28 kHz in the pulses from Cephalotaxus, Capsicum, and Artemisia, respectively. In addition, peaks close to integral multiples of *f*_p(axial)_ are observed. Analysis of pulses from the rest of the plant species shows similar trends (Figure S3-S11 and Table 1).

**Figure 4:**
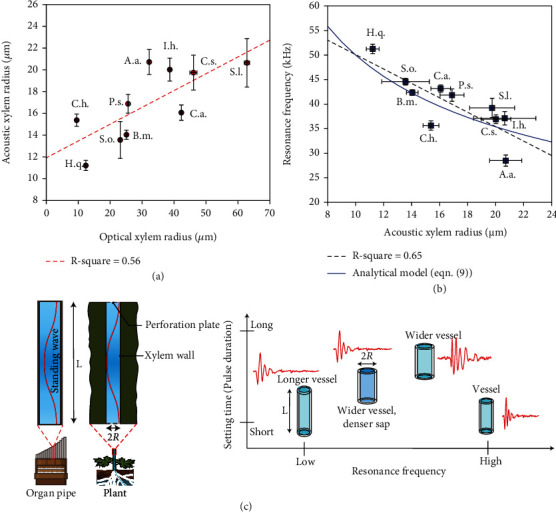
Acoustic model validation for xylem radius and ultrasound resonance frequencies. (a) Linear relationship (red-dashed line) between acoustic and optical xylem vessel radius for each species. Three shoot samples were measured for each species for sound recording and optical microscopy. Red circles represent the mean (over pulses from all samples) and the error bars represent the standard error. (b) Inverse relationship (black-dashed line) between the extracted resonance frequency and the acoustic xylem radius. Blue squares represent the mean, and the error bars represent the standard error. The solid blue curve indicates the model predicted trend between resonance frequency and vessel radius, assuming constant values of *L* = 1 mm, *h* = 1 *μ*m, and *E* = 0.2 GPa in equation (9). (c) Schematic illustrating the analogy between standing pressure waves in the xylem vessels and those in an organ pipe. Also shown are the predicted qualitative effects of changing vessel element length, radius, and sap density/viscosity on the resonance frequency and settling time of emitted ultrasound pulses.

The resonance frequency (*f*_L_) is calculated from *f*_p(axial)_ (see Materials and Methods). Note that the two values differ due to the high damping (small *τ*_s_) in the sound pulse. According to our acoustic pipe model (see equation (9) in Materials and Methods), *f*_L_ decreases with increasing vessel radius *R*. In Figures 3(e)–3(h) and Figures S3-S11, we observe a similar trend in in the resonance frequencies extracted from the recorded ultrasound pulses in all the ten plant species. The ultrasound methodology is thus validated for multiple plant species, showing the link between the vessel radii and the settling time of the ultrasound pulses. The acoustic *R* shows a linear relationship (*R*^2^ = 0.56) with the optically observed vessel radius (see Figure 4(a)). We also observe that the sample mean of the resonance frequencies in each species shows an inverse relationship with the acoustic radius (*R*^2^ = 0.65), as shown in Figure 4(b), hence corroborating our model. Figure 4(b) also illustrates the model predicted trend between *f*_L_ and *R* according to equation (9) in an ideal case, where a fixed value is assigned to the each of the parameters *L*, vessel wall thickness *h*, and Young's modulus of elasticity *E*. These parameters are discussed further in the next paragraph. Overall, our model makes an analogy between the xylem vessel elements and flutes in an organ pipe, which communicate their physical state via the pitch and duration of their own “music” (see Figure 4(c)). To summarize, Figure 4(c) further illustrates the predicted qualitative effect of *R*, *L*, and sap density on the time and frequency characteristics of the emitted acoustic pulse. After having studied the relation between the ultrasound waveform and the xylem vessel radius, we now address the connection to Young's modulus and xylem vessel element length.

### 2.3. Young's Modulus of *H. quercifolia* Stems and Xylem Vessel Element Length

**Figure 5:**
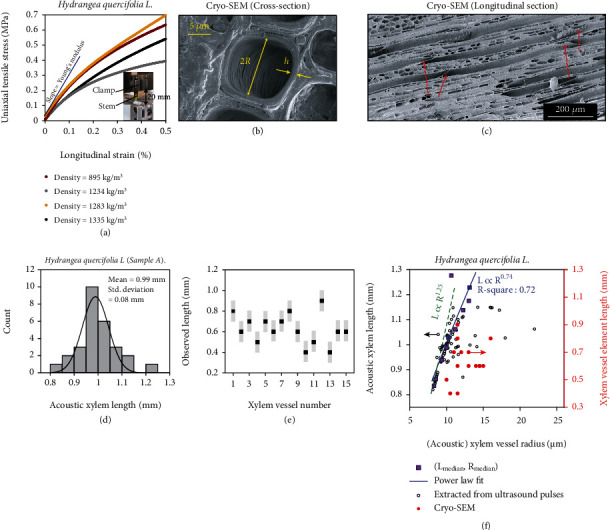
Young's moduli of stem and extraction of xylem vessel element lengths. (a) Measured stress-strain curve for freshly cut stem segments of Hydrangea with mass densities indicated in the legend. The blue line represents the linear fit of a stress-strain curve at low strain, the slope of which yields the Young's modulus in accordance with Hooke's law. The inset shows the photograph of the set-up for uniaxial tensile loading of the stem. (b) Cryo-SEM image of the transverse section of a stem sample showing the diameter (2*R*) and the wall thickness (*h*) of a xylem vessel. (c) Example cryo-SEM image of the longitudinal section of a Hydrangea stem segment, showing the structure of individual vessel elements terminated by scalariform perforation plates (marked by red arrows). Observed length of each element is in the range 600 *μ*m–900 *μ*m. (d) Histogram showing the extracted xylem vessel element lengths in stem sample A, extracted via the acoustic model. The black curve represents a unimodal Gaussian fit. (e) Scatter plot showing the observed length of xylem vessel elements via cryo-SEM technique. The grey bars indicate the upper and lower bounds due to the slant length of the perforation plates along the longitudinal axis (~100 *μ*m). (f) Scatter plot of model-extracted (acoustic) xylem vessel element length (*L*) and radius (*R*) corresponding to each analysed ultrasound pulse (black circles). Data from all three Hydrangea stem samples are merged here. The radii were obtained from the settling time of the ultrasound pulses, while the lengths were obtained from the resonance frequency of the sound pulses and by measuring Young's modulus. The data points are classified into bins of *L* with intervals of 0.5 mm. In each bin, the median *L* and *R* are calculated (pink squares) and fitted with a power law function (blue line). The green-dashed line indicates the predicted *L*‐*R* dependency in accordance with Darcy-Weisbach equation for fluid flow, combined with mechanical failure of the xylem vessel (see Materials and Methods). Red circles: observed vessel element lengths and radius in Hydrangea via cryo-SEM technique.

In our model, *f*_L_ depends on the speed of sound in bulk water *v*_l_, the vessel element length *L*, wall thickness *h*, and the Young's modulus of elasticity *E*. Young's modulus quantifies the stiffness of a solid against mechanical stress. It is defined as the amount of stress along a given direction, required to produce unit relative change in its dimension. To estimate *L* of the resonating element of the xylem vessel, we need to obtain *h* and *E*. We determined *E* of stem segments cut from the same plant (see Materials and Methods) and from shoots similar in age and size. For this, we measured the stress-strain curves via uniaxial tensile loading (Figure 5(a)). The mean mass density per stem segment was also estimated from the measured weights and dimensions. The linear slope of the stress-strain curve (Figure 5(a)) at small values of strain (≈10^−4^) yields the value of *E*, which is extracted to be 0.2 ± 0.1 GPa for fresh (hydrated) stem samples (Figure S12). For dry stem samples, *E* > 0.6 GPa is obtained. We observe an overall decline in *E* with increasing mass density. This indicates that the water content dominates the variations in *E*. This agrees with an earlier empirical model [26], where the dependence of *E* on the relative water content in the xylem is taken into account. We observed *h* to be ~1 *μ*m via scanning electron cryomicroscopy (see Figure 5(b)).

We calculate *L* using *h* ≈ 1 *μ*m and *E* = 0.2 ± 0.1 GPa in equation (9) (see Materials and Methods). The histogram of *L* is extracted from the axially recorded ultrasound pulses for stem samples. For Hydrangea sample A, *L* is obtained to be 0.99 ± 0.08 mm (see Figure 5(d)). Similar values are obtained for samples B and C (Figure S13). This highlights the reproducibility of our method and the similarity of the recorded ultrasound pulses.

Next, we validate the assumption that *L* represents the actual length of xylem vessel element. We extract the mean xylem vessel length (a vessel contains several vessel elements) using latex paint staining [32], by counting the number of stained vessels on transverse cross-sections of the stem. These counts decrease exponentially with the distance [35] from the lower end of the stem at which the paint was taken up (Figure S14). Xylem vessel lengths are found to be in the range ~12–17 mm, meaning that *L* extracted using this method is much larger than the *L* extracted from the ultrasound pulses (~1 mm, Figure 5(d)). This is because the latex paint molecules cannot penetrate the fused ends but can pass through the perforation plates between adjacent vessel elements [35]. Subsequently, we observed individual vessel elements in longitudinal sections of stem samples using scanning electron cryomicroscopy (Figure 5(c)). The observed length ranges from 0.5 to 0.9 mm for individual xylem vessel elements (Figure 5(e)). Thus, *L*, as obtained from our acoustic model, is a good estimate of the length of individual vessel elements.

### 2.4. Relationship between *L* and *R* in *H. quercifolia*

Our method of analysing ultrasound emissions enables us to generate a set of length versus radius data for xylem vessel elements within a given stem segment. To our knowledge, a little has been reported on the relationship between radius *R* and length *L* of single vessel elements [36]. Prior studies across several angiosperm species have reported on the theoretical and experimental relationships between *R* and total length of xylem vessels *L*_v_ [24, 37], expressed as *L*_v_ ∝ *R*^*a*^. The scaling exponent a has been reported [37] to take experimental values between 1.0 and 3.0, whereas optimal values such as 1.5 and 2.5 have been predicted theoretically under considerations of morphological traits of xylem vessels and competing trends to maximize hydraulic conductance and to minimize susceptibility to cavitation.

We observe that in a single plant (Hydrangea), *L* scales as *R*^0.74^ (see Figure 5(f)). Basic fluid and structural mechanics help us in predicting an upper bound on *L*‐*R* dependency. In plants of height within ~1 m, transpiration pull is the governing force of water ascent through xylem vessels, which creates a gradient in the hydrostatic pressure along the vascular column. With a constant volume flow rate of water through the series-connected vessel elements (continuity), the pressure drop along a length *L* can be obtained from the Darcy-Weisbach equation (see Materials and Methods). Furthermore, a vessel element can withstand a maximum pressure drop to avoid rupture [38]. This critical pressure is also a function of both *L* and *R* (see Materials and Methods). Combining the two dependencies, we derive that *L*_crit_ ∝ R^1.25^, where Young's modulus and wall thickness are assumed to be constants. This reasoning provides us an upper bound on the scaling exponent from a purely mechanical viewpoint.

## 3. Discussion

We have shown a method for using ultrasound emissions from drought-stressed plant stems to extract and monitor the geometry and viscoelasticity of xylem vessels. In this section, we interpret the results and discuss the applicability of the method to monitor the vascular physiology of plants.

### 3.1. Xylem Vessel Radius

We have shown that by modelling the xylem vessel as cylindrical acoustic resonator, the radius *R* can be extracted from the settling time of the ultrasound pulse, resulting in comparable values as those obtained from common microscopy techniques for multiple species. Using a range of vascular plant species, with varying vessel radii, we validate the dependency of *τ*_s_ on *R* (Figure 4(c)). We also show that our method works for the gymnosperm Cephalotaxus, containing tracheids, instead of xylem vessels, as the hydraulic conduits. In most experiments, optically determined xylem vessel radii were found to be bigger than the acoustically determined radii, the deviation varying from species to species (Figure 4(a)). We attribute this mainly to the assumption of a constant dynamic viscosity of xylem sap *η*_l_. In practice, *η*_l_ depends on ambient temperature and concentration of dissolved nutrients [39]. Moreover, water close to the sap-wall interface is held with adhesive forces and thus has a slightly higher dynamic viscosity [40]. Note that the solid walls of the xylem vessels also possess shear or extensional viscosity [41]. This means that elastic forces arise in them as a response to elongation, compression, or shear stresses. Shear viscosity is a property of solids to resist a change in deformation (shear rate). This additional viscosity likely sets an upper bound on *τ*_s_ and *R*, beyond which the quantitative agreement between optical and acoustic radii likely deteriorates. This is also evident from Figures 2(k) and 2(l), SI7, and SI11 where we observe a larger deviation between the mean acoustic and optical radii for Capsicum, Artemisia, Cucumis, and Solanum, respectively.

### 3.2. Xylem Vessel Element Length (*L*) and Young's Modulus (*E*)

The xylem vessel element length *L*, extracted from the ultrasound pulses (Figure 5(d)) consistently exceeds the physical length (via SEM; Figure 5(e)) by ~0.3 mm (~30%). We attribute this to two factors. Firstly, the perforation plates serve as nonrigid and leaky boundaries (not accounted for in the model), due to which the standing waves penetrate beyond the physical length of a single vessel element. Secondly, the uniaxial tensile loading measurements that we performed (Figure 5(a)) on stems provide an overestimation of the xylem Young's modulus. This is due to the presence of stiffer sclerenchyma and collenchyma tissue [42], with Young's moduli exceeding ~1 GPa [43], close to the circumference of the stem. Hence, as a corollary to our analysis, one can alternatively obtain the effective Young's modulus by an independent measurement of the xylem vessel element length via microscopy. Xylem cells differentiate very early during the growth of a plant [44], subsequently growing to their maximum lengths before maturing (dying) [45] to become hydraulically active vessel elements. Thus, once the vessel element length is determined via microscopy techniques for a given plant, the Young's modulus can then be continuously and noninvasively monitored to diagnose variations in water content [46], ageing, or even pathogen-induced occlusions within the xylem [47, 48].

### 3.3. Impact and Scope

The state of the art for determining xylem vessel properties is largely centered on cutting stems and examining them under a microscope, which comes with obvious drawbacks to speed and scalability. Using methods like latex paint staining and scanning electron microscopy to monitor xylem vessels is time-consuming (~10 hours–1 day), and also it cannot be applied real time to living plants. Recently, X-ray microtomography was recommended [49] to monitor xylem embolisms and hydraulic vulnerability. However, to date, this method is expensive and not suitable for field applications. Ultrasound allows for monitoring xylem vessels with relatively inexpensive apparatus [50] with a measurement time under 1 minute. The development of an ultrasound actuation method for the standing waves in plants' xylem vessels would enable direct in vivo applications in, e.g., indoor farms and greenhouses. In contrast to lab-based microscopy, the ultrasound technology can offer nondestructive and continuous measurements while being well accessible to nonspecialists. Note that although we have recorded the ultrasound from cut shoots to accelerate the stress response, such incision is not mandatory for sound to be emitted during drought stress as already shown in literature [10, 13, 14, 18].

The presented methodology establishes a link between geometrical properties of xylem vessels and the recorded ultrasound emissions of plants across multiple species (Figure 4). We observed that vessel radii (*R*) exhibit a clear statistical trend with pulse settling time and with the recorded ultrasound frequency, which is a measure for the xylem vessel element length (*L*). Note that although earlier studies on *L* and *R* were reported in literature [30, 50, 51], those were obtained only across different species by either destructive microscopy or X-ray microtomography. We show, for the first time, the rapid determination of both *R* and *L* in a single plant (Figure 5(f)), which enables studies on their physical relationship. This could equip plant physiologists with a convenient tool to conduct further studies about the vasculature in relation to plant hydraulics and its effect on phenotype or age of the plant. While determining *R* aids in studying embolism and hydraulic transport capacity [8], *L* has also been reported to correlate with the photosynthesis rate and water use efficiency of certain species [36]. Further, our method can also be extended to study and possibly distinguish between the various sources of acoustic emissions from the plant based on the pulse characteristics.

We foresee applications of our method to a multitude of plant species with varying vessel dimensions and viscoelasticity. Although only herbaceous and shrub-like dicot plants were examined in this work, our method might also apply to monocots, since some of these have a similar vessel structure as dicots. However, this remains to be tested. Further, from a scientific viewpoint, the method can be combined with advanced techniques such as micro-CT and acoustic microscopy [9] to provide visual evidence of the sound emissions and study the influence of vessel morphology (e.g., type of perforation plates) on the nature of bubble formation. Our method was, so far, only applied to measurements in a lab environment, and we characterized the shoots only at the onset of drought stress. Nonetheless, it opens the route to monitor possible changes in the ultrasound pulse characteristics over a prolonged dehydration period of a plant. The method also enables further in vivo studies on the mechanical resonances of a plants' vascular tissue via external acoustic excitation. In turn, this provides a noninvasive method for rapid phenotyping. Crops could be selected for breeding based on their xylem vessels and thus based on their response to drought and/or susceptibility to vascular wilt pathogens [44, 45, 52, 53]. Drought stress impacts the viscoelasticity of the vascular tissue. Correlation between vessel radius and drought stress has been reported in poplar [54] and apple trees [55]. Both radius and viscoelasticity could change the intensity and settling time of the emitted ultrasound pulses. Further, pathogens within the xylem vessels are known to cause intravascular occlusions and can affect the sucrose/nutrient concentration in the sap, e.g., in olive trees [56], which can likely change the kinematic viscosity of the xylem sap, thereby opening new routes of research in plant-sound relations for noninvasive diagnoses. We therefore call for such further studies.

Lastly, from the viewpoint of a complete sensor system, the presented methodology only uses Fourier transforms and envelope detection. These are standard signal processing functions, which can be implemented in commercial integrated chip technology. This will help with future development of low-cost and compact tools for monitoring plant stress. This will in turn boost climate-smart agriculture and indoor farming by providing farmers with new tools for optimal irrigation strategies and early disease detection. The presented methodology provides a new outlook on plants “talking” during drought stress and presents ultrasound sensing as an inexpensive technique for rapid, noninvasive, and in vivo characterization of plant vasculature.

## 4. Materials and Methods

### 4.1. Plant Material and Shoot Sample Preparations

Three plants each from ten plant species, namely, *Hydrangea quercifolia* (H.q.), *Artemisia annua* (A.a.), *Begonia maculata* (B.m.), *Plectranthus scutellarioides* (P.s.), *Impatiens hawkeri* (I.h.), *Salvia officinalis* (S.o.), *Cucumis sativus* (C.s.), *Capsicum annuum* (C.a.), *Cephalotaxus harringtonia* (C.h.), and *Solanum lycopersicum* (S.l.), were obtained. The Artemisia and Capsicum plants were grown in a greenhouse, whereas the Solanum and Cucumis plants were grown in a climate chamber. The remaining species were procured from a commercial garden center and moved to the laboratory within 1 hour. All plants were well watered to avoid onset of drought stress prior to our experiments. Three mature shoot samples, one per plant, were cut, keeping the leaves intact, and immediately placed in tap water (Figure S15) to prevent embolism in the xylem vessels at the cut-end. For Hydrangea, a 60-70 mm long stem segment was cut from each shoot and trimmed (i.e., without leaves and petioles) under water to prevent air entry and blockage. The segments were roughly cylindrical, with a cross-section diameter of ~5-6 mm, and were used for vessel staining (with latex paint) and optical microscopy. The reason behind using latex paint infiltration for Hydrangea shoots was to eventually estimate mean vessel lengths via a counting method described in a later subsection. The rest of the sample was left intact to measure ultrasound emissions. For the remaining nice species, first a 60-70 mm long shoot was cut under water (with leaves and petioles) for ultrasound recording. From this shoot, a 30 mm long stem segment was subsequently cut and trimmed for optical microscopy.

### 4.2. Recording Ultrasound Pulses and Signal Processing

The shoot samples were taken out of water, dried using tissue paper, and left on the bench for air drying, resulting in accelerated drought stress (Fig. SI15). A M500-USB ultrasound microphone, with a reliable detection window between 10 kHz and 150 kHz, from Pettersson Elektronik AB (Uppsala, Sweden), was placed first in the axial (~2 mm from the cut-face of stem normal to the cross-section) and then in the radial (on the cylindrical surface of the stem) directions (Figure 1(a)) to record the ultrasound bursts at a sampling rate of 500 kHz for a continuous time duration of 70 seconds, at room temperature (298 K). The sensor consists of a piezoelectric material which produces an electrical voltage proportional to the pressure of the incident sound wave. Influence of background noise and spurious signals was minimized by setting a pulse detection threshold of 0.005 a.u. at the microphone output. From the time-domain waveforms, the pulse envelope was obtained with the built-in “envelope ()” function in MATLAB, which returns the upper and lower envelopes of the input sequence, as the magnitude of its analytic signal. The analytic signal of the input sequence was found using the Hilbert transform. The peak of the envelope curve was determined, and the decreasing part of the envelope curve was stored, which was subsequently fitted with the exponential function exp (−*t*/*τ*_s_) using the least squares method, where the variable *t* denotes time. This yielded the settling time *τ*_s_. The frequency spectra of the measured signals were obtained via a 250-point discrete Fourier transform, spanning a time frame of 1.5 ms. Due to the low intensity of the emitted sound, the spectra are shown until 150 kHz beyond which the signal merges with the noise floor of the sensor (-80 dB). The raw data was then postprocessed and analysed in MATLAB R2018b (MathWorks, Massachusetts, USA).

### 4.3. Analytical Model for Longitudinal Vibrations

We modelled the xylem vessel element as a cylindrical pipe of radius *R*, and effective length *L* sustaining longitudinal standing waves [57, 58] in the water of density *ρ*_l_ whose resonance frequencies depend on the mode order *m* and the longitudinal speed of sound in the pipe *v*_eff_. The resonant frequency of the *m*^th^ order (*m* = 1, 2, ⋯) is given by

(1) $$\begin{array}{c} f_{\text{m}}=\left(\frac{\text{m}}{2}\right)\frac{v_{\text{l}}}{L}, \end{array}$$

where *v*_l_ is the speed of sound in the liquid (~1482 m/s in bulk water at 20°C). We denote the fundamental resonance frequency (*m* = 1) as *f*_L_ in the rest of this section. In practice, equation (1) cannot be applied directly because in a real pipe with an elastic wall, sound propagates at a slower speed than that in the bulk liquid. If the walls of the pipe have a nonzero acoustic thickness *h* and finite Young's modulus *E*, then the effective speed of sound is given by [59, 60]

(2) $$\begin{array}{c} \left(\frac{1}{v_{\text{eff}}^{2}}\right)=\left(\frac{1}{v_{\text{l}}^{2}}\right)+\rho_{\text{l}}\beta_{\text{xylem}},\text{ where }\beta_{\text{xylem}}=\left(\frac{2R}{hE}\right), \end{array}$$

where *β*_xylem_ is known as the cross-sectional compressibility and *ρ*_l_ = 996 kg.m^−3^ is the mass density of water. Thus, *v*_l_ is replaced by *v*_eff_ in equation (1).

These sound waves (expected to be dominant in the axially recorded ultrasound) undergo damping primarily due to the dynamic viscosity of water [61] *η*_l_ in the xylem. The resulting time-domain response of the resonating pipe can be described using a lumped circuit model consisting of acoustic inductance (*L*_a_), capacitance (*C*_a_), and resistance (*R*_a_), analogous to an electrical *L*‐*C*‐*R* circuit, where voltage and current are replaced by pressure and flow rate, respectively. *L*_a_ is a consequence of the kinetic energy in the water, while *C*_a_ arises due to the compressibility of water. *R*_a_ leads to energy dissipation and can be obtained from Poiseuille's law for capillary flow [41, 57]. The three lumped parameters can be expressed as

(3) $$\begin{array}{c} L_{\text{a}}=\frac{L.\rho_{\text{l}}}{\pi .R^{2}},\\ C_{\text{a}}=\frac{L.\pi .R^{2}}{\rho_{\text{l}}.v_{\text{l}}^{2}},\\ \;R_{\text{a}}=\frac{8.\eta_{\text{l}}.L}{\pi .R^{4}}, \end{array}$$

where *η*_l_ = 8.9 × 10^−4^ Pa.s is the dynamic viscosity of water. By describing the circuit as a linear 2^nd^ order differential equation, we obtained the damping ratio *ζ*, envelope settling time *τ*_s_ (the time needed for the amplitude to decrease by a factor of “*e*”), and the driving frequency *f*_d_ as

(4) $$\begin{array}{c} \zeta =\frac{R_{\text{a}}}{2}\sqrt{\frac{C_{\text{a}}}{L_{\text{a}}}}=\frac{4.\eta_{\text{l}}.L}{\rho_{\text{l}}.v_{\text{l}}.R^{2}}, \end{array}$$

(5) $$\begin{array}{c} \tau_{\text{s}}=\frac{1}{\zeta .f_{\text{L}}}=\left(\frac{\rho_{\text{l}}}{4\eta_{\text{l}}}\right).R^{2}, \end{array}$$

(6) $$\begin{array}{c} f_{\text{d}}=f_{\text{L}}\sqrt{1-\zeta^{2}}. \end{array}$$

The lumped model is valid as long as the dimensions *L* and *R* are smaller than the acoustic wavelength (~1-10 cm in water). Noting that *f*_d_ is the same as the observed *f*_p(axial)_ in the ultrasound pulses, *ζ* is obtained by combining equations (5) and (6) as

(7) $$\begin{array}{c} \zeta =\frac{1}{\sqrt{1+{\left(f_{\text{p}\left(\text{axial}\right)}.\tau_{\text{s}}\right)}^{2}}}. \end{array}$$

And the acoustic xylem radius was obtained by rearranging equation (5):

(8) $$\begin{array}{c} R=\sqrt{\frac{4.\eta_{\text{l}}.\tau_{\text{s}}}{\rho_{\text{l}}}}. \end{array}$$

Combining equations (1) and (2), the effective length *L* was obtained as

(9) $$\begin{array}{c} \frac{1}{L^{2}}=\frac{4f_{\text{L}}^{2}}{{\text{m}}^{2}v_{\text{eff}}^{2}}=\frac{4f_{\text{L}}^{2}}{{\text{m}}^{2}}\left[\frac{1}{v_{\text{l}}^{2}}+\frac{2\rho_{\text{l}}R}{h}.\left(\frac{1}{E}\right)\right]. \end{array}$$

### 4.4. Scanning Electron (Cryo-)microscopy

SEM was performed in order to observe vessel element lengths and wall thickness in greater detail. Transverse sections from Hydrangea stems were made using a razorblade. The cross-section was left on filter paper for 1-2 minutes to remove most of the adhering water. Thereafter, the section was fixed to a sample holder using Tissue-Tek. The sample was frozen by plunging the sample holder into liquid nitrogen. Subsequently, the sample was transferred to a cryopreparation chamber (Leica Microsystems, Wetzlar, Germany) under vacuum where it was kept at -90°C for 3 minutes to remove ice from the surface (freeze etching to remove water vapor contamination). While still under vacuum, the sample was coated with 12 nm of tungsten and transferred using a VCT100 shuttle (Leica) to a field emission scanning electron microscope (Magellan 400 from FEI, Oregon, USA). The samples were analysed at 2 kV and 13 pA at -120°C.

Longitudinal sections were made by carefully cutting through the region that contains the xylem vessels. The rest of the sample preparation was identical.

### 4.5. Uniaxial Tensile Loading for Young's Modulus Determination

Young's modulus measurements were done to substitute values for the elasticity parameter in our model relating vessel element length and the resonance frequency. Multiple stem segments of lengths in the range of 4-7 cm were cut and mounted vertically between two clamps of a tensile testing machine (Z005; Zwick/Roell, Ulm, Germany; inset of Figure 5(a)). The initial prestrained length (*l*_0_) is equal to the vertical separation between the clamps and was kept as 20 mm. The uniaxial stress was calculated as the tensile force applied by the equipment divided by the average cross-section area of the stem segment. The longitudinal strain was calculated as the change in stem length per unit initial length (Δ*l*/*l*_0_). Young's modulus *E* was then extracted as the slope of the linear part of the stress-strain curve (Figure 5(a)) at small values of strain (≈10^−4^). The average mass density of each sample was also calculated from measured weight and volume just before tensile loading. The weights were measured with a Scaltec SBC 33 precision balance (Scaltec Instruments GmbH, Göttingen, Germany), while the dimensions were measured with a standard Vernier calliper with a resolution of 0.1 mm. Note that the measurement error for elastic moduli and mass density (~ 20%) is predominantly due to error propagation from length and diameter measurements.

### 4.6. Vessel Staining and Optical Microscopy

For *H. quercifolia* stem samples, latex paint infiltration was used for vessel staining. This was done to estimate the mean xylem vessel length in the sample via vessel counting, in addition to observing the xylem vessel radii via optical microscopy. An aqueous solution 1% (*v*/*v*) suspension of red latex paint was left standing for at least 24 hours to allow large particles to settle at the bottom. The supernatant was subsequently transferred to a glass container and degassed. The stem segments were mounted vertically over the glass container, with one end immersed in the paint and the other end tightly inserted into a plastic tube connected to a suction pump (Figure S15) which applied a pressure difference of 400 mbar. The stem-tube junction was taped and smeared with Vaseline to prevent air leakage. As the solution was sucked through the stem for 12 hours, the paint remained confined in one xylem vessel (macromolecules in the paint cannot move through the bordered pits of xylem vessels), while the clear water was conducted through the entire stem. Subsequently, the stem samples were sliced with a blade at intervals of 5 mm. The number of painted vessels was then counted on each face of the cut slices from images (magnification of 200x) captured by a VHX digital microscope from Keyence.

An exponential relationship was observed [62] for the number of continuous xylem vessels at varying lengths of a stem segment. Typically, it is observed that longer vessels are also wider [37]. The complex relationship between xylem radius and length in a plant is largely affected by a trade-off between hydraulic conductance (increases with increasing *R* and decreasing *L*) and vulnerability to cavitation [63] (increases with increasing *R* and *L*). The xylem vessel length has the following probability distribution function [62, 63]:

(10) $$\begin{array}{c} P\left(x\right)=x.\lambda_{\text{xylem}}^{2}\exp\left(-\lambda_{xylem}x\right), \end{array}$$

where the most probable vessel length is given by *λ*^−1^_xylem_, while the mean and standard deviation are, respectively, given by 2 *λ*^−1^_xylem_ and 1.414 *λ*^−1^_xylem_. Equation (10) is based on the assumption that xylem vessels have, for every additional unit length, a similar chance to terminate [63]. Thus, we describe the length distribution of a vessel population. Starting from a chosen reference position *x* = 0, the number of vessels *N* with length *L* ≥ *x* is given by

(11) $$\begin{array}{c} N=N_{0}\exp\left(-\lambda_{\text{xylem}}x\right). \end{array}$$

For the remaining nine species, a 30 mm long stem was taken from each shoot sample, and slices were made by either blades or a microtome. The slices were stained by 1% (*v*/*v*) toluidine blue. These slices were then placed under an optical microscope (Leica, MZ Apo, Germany) for inspection at varying magnifications. The xylem radii were measured from the cross-section images with ZEISS ZEN 3.4 (blue edition) software (Carl Zeiss Microscopy GmbH, Germany). The best three stained slices were chosen for each species, and in total, 72 xylem vessels were measured from each shoot sample.

### 4.7. Darcy-Weisbach Equation and Critical Pressure in Xylem Vessel

The Darcy-Weisbach equation is an empirical relation that relates the pressure drop *p* along a given length *L* of a viscous and incompressible fluid flowing through a conduit of radius *R* as

(12) $$\begin{array}{c} \text{p}=\frac{8\eta_{\text{l}}\text{ Q L}}{\pi {\text{R}}^{4}}, \end{array}$$

where *Q* is the volumetric flow rate and *η*_l_ is the dynamic viscosity of the fluid (water).

From the viewpoint of mechanical rupture/failure, a biomechanical model was reported [38] where a vessel element is treated as a cylindrical shell under hydrostatic pressure of length *L*, radius *R*, uniform wall thickness *t*, and isotropic homogeneous Young's modulus *E*. For cylinders with *L*/(*R*.*t*)^0.5^ > 4 (applicable for xylem vessel elements), the critical hydrostatic pressure *p*_crit_ can be found from Batdorf's approximate formula [38, 64] as

(13) $$\begin{array}{c} p_{\text{crit}}=\frac{0.92\;t^{5/2}\;E}{L.R^{3/2}}. \end{array}$$

So, to prevent mechanical failure, *p* < *p*_crit_. Substituting the above expressions and rearranging the terms, we obtain

(14) $$\begin{array}{c} L<\left(\sqrt{\frac{0.92\;\pi \;t^{5/2}E}{8\;\eta_{l}Q}}\right).R^{5/4}. \end{array}$$

## Figures and Tables

**Table 1:** Summary of extracted vessel parameters for the plant species. List of resonance frequency and acoustic xylem radius obtained via the ultrasound analysis, and those obtained by direct optical microscopy, for the ten plant species studied in this manuscript. In the columns of optical and acoustic radius, *N* denotes the number of vessels and acoustic pulses measured, respectively.

Species name	Source	Optical vessel radius (mean ± SE) (*μ*m)	Acoustic vessel radius (mean ± SE) (*μ*m)	Peak resonance frequency (kHz)
Hydrangea quercifolia (H.q.)	Commercial garden center	12.4 ± 0.3 (*N* = 53)	11.2 ± 0.5 (*N* = 91)	51.2 ± 1.0
Artemisia annua (A.a.)	Greenhouse (Wageningen University and Research)	32.2 ± 0.2 (*N* = 356)	20.7 ± 1.1 (*N* = 153)	28.5 ± 1.1
Begonia maculata (B.m.)	Commercial garden center	25.1 ± 0.2 (*N* = 216)	14.0 ± 0.4 (*N* = 415)	42.4 ± 0.6
Capsicum annuum (C.a.)	Greenhouse (Wageningen University and Research)	42.3 ± 0.3 (*N* = 213)	16.1 ± 0.7 (*N* = 187)	43.2 ± 0.7
Cephalotaxus harringtonia (C.h.)	Commercial garden center	9.5 ± 0.4 (*N* = 216)	15.4 ± 0.6 (*N* = 126)	35.6 ± 0.9
Cucumis sativus (C.s.)	Climate chamber (Wageningen University and Research)	46.1 ± 1.1 (*N* = 217)	19.8 ± 1.6 (*N* = 78)	39.2 ± 2.0
Impatiens hawkeri (I.h.)	Commercial garden center	38.7 ± 0.4 (*N* = 216)	20.0 ± 1.0 (*N* = 263)	36.9 ± 0.9
Plectranthus scutellarioides (P.s.)	Commercial garden center	25.6 ± 0.2 (*N* = 216)	16.9 ± 0.9 (*N* = 172)	41.8 ± 1.3
Salvia officinalis (S.o.)	Commercial garden center	23.1 ± 0.2 (*N* = 216)	13.6 ± 1.7 (*N* = 294)	44.6 ± 0.7
Solanum lycopersicum “Merlice” (S.l.)	Climate chamber (Wageningen University and Research)	62.8 ± 0.9 (*N* = 216)	20.6 ± 2.2 (*N* = 151)	37.1 ± 1.4

## Data Availability

The data that support the findings of this study are available from the corresponding author upon reasonable request.
