# Supplementary material for: Ultrasound Pulse Emission Spectroscopy Method to Characterize Xylem Conduits in Plant Stems
Source: Research (Wash D C). 2022 Sep 13;2022:9790438. doi: 10.34133/2022/9790438 (PMC9513830; doi:10.34133/2022/9790438)
Supplement: Supplementary materials — Supplementary material for this article is available. Figures S1–S15. [file 9790438.f1.docx]

Supplementary Information for

**Ultrasound Pulse Emission Spectroscopy Method to Characterize Xylem Conduits in Plant Stems**

Satadal Dutta,^*^, Zhiyi Chen, Elias Kaiser, Priscilla Malcolm Matamoros, Peter G. Steeneken, Gerard J. Verbiest^*^

*Corresponding authors. Email: [s.dutta-1@tudelft.nl](mailto:s.dutta-1@tudelft.nl), [g.j.verbiest@tudelft.nl](mailto:g.j.verbiest@tudelft.nl)

**This section includes:**

Figs. S1 to S15


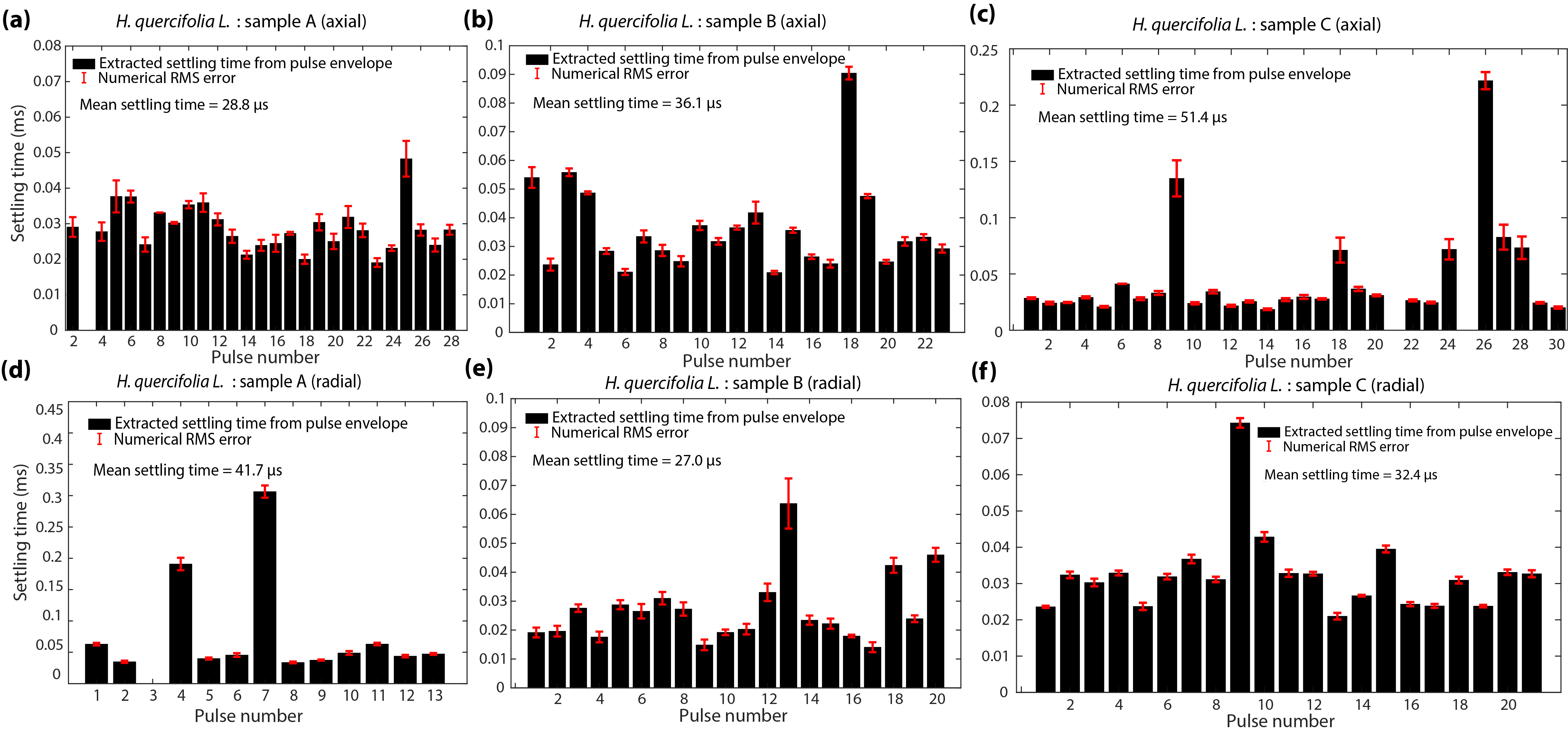


Fig. S1.

**Ultrasound pulse settling times in *Hydrangea quercifolia***. **(a), (b), (c)** Bar plots of the extracted amplitude settling times (*τ*_s_) for the ultrasound pulses recorded from Hydrangea shoot samples A, B, and C in the axial direction, respectively. Settling time is obtained with an exponential fit of the pulse envelope (**Fig. 1C, 1D,** see Materials and Methods). The red bars indicate the error margin in the numerical fit routine. **(d), (e),(f)** Bar plot of the extracted amplitude settling times (*τ*_s_) for the ultrasound pulses recorded from Hydrangea stem samples A, B, and C in the radial direction, respectively.

Fig. S2.


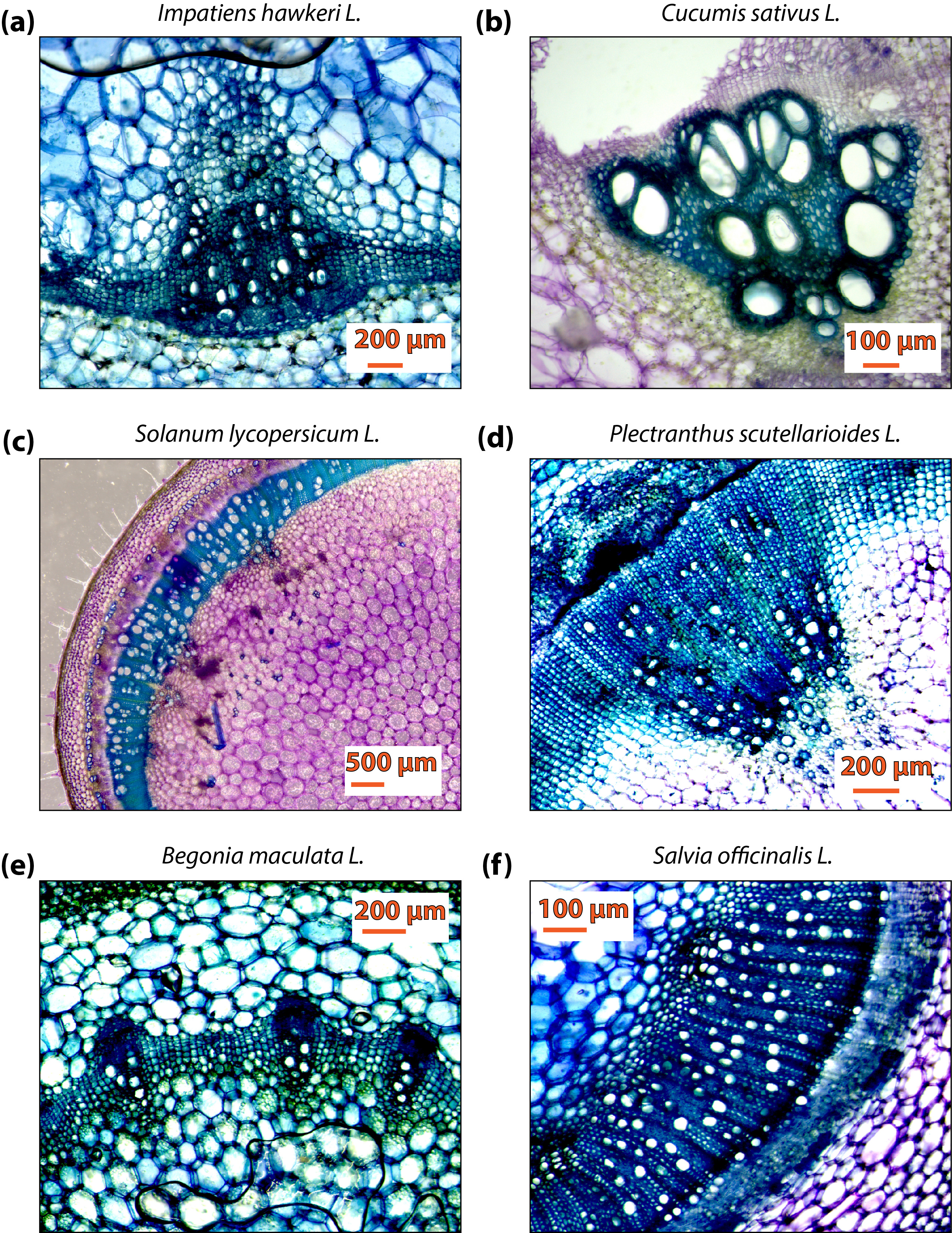


**Optical microscopy of xylem vessels.** **(a)-(f)** Optical micrographs of transverse section of stem samples of *Impatiens hawkeri*, *Cucumis sativus*, *Solanum lycopersicum*, *Plectranthus scutellarioides*, *Begonia maculata*, and *Salvia officinalis*, respectively. All images were captured with a VHX digital microscope (Keyence International NV/SA, Mechelen, Belgium).


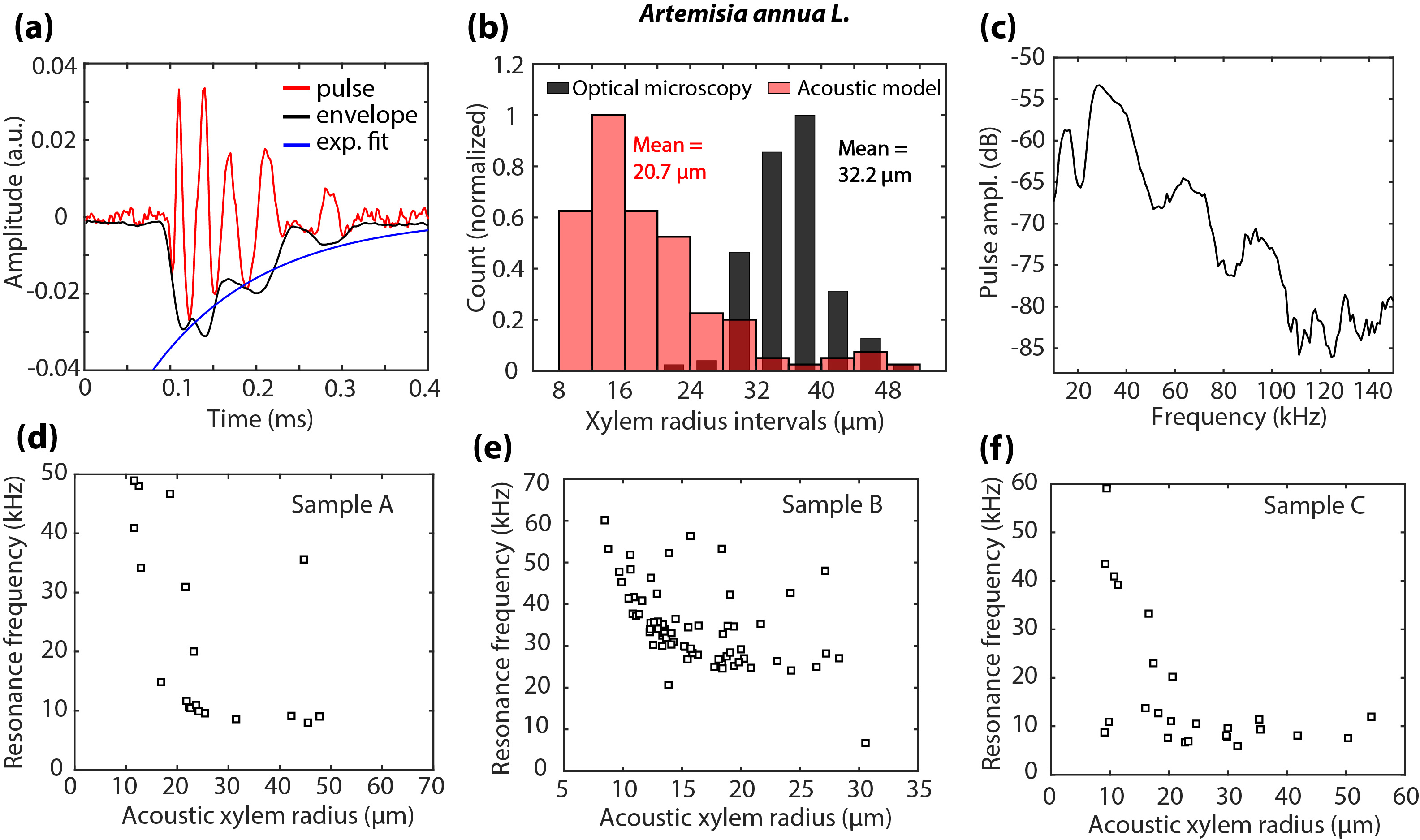


**Fig. S3**.

**Ultrasound pulse analysis for *Artemisia annua***. **(a)** Zoomed-in time-domain waveform of an example ultrasound pulse from Artemisia stem, recorded axially (**Fig. 1a**). The recording is done with a M500-USB microphone from Pettersson Elektronik AB. Black curves represent the amplitude envelope, and the blue curve represents the exponential fit of the pulse envelope (**Fig. 1c, 1d,** see Materials and Methods). **(b)** Histogram showing the model-extracted xylem radii (in red), and that of the observed xylem radii (in black) obtained via optical microscopy. **(c)** Fourier transform of the example ultrasound pulse shown in (a) showing the characteristic peak frequencies. **(d)-(f)** Model-extracted resonance frequency versus acoustic xylem radius for sound pulses from stem samples A, B and C, respectively. Resonance frequency is obtained from the peak frequency of highest amplitude in the recorded pulses.


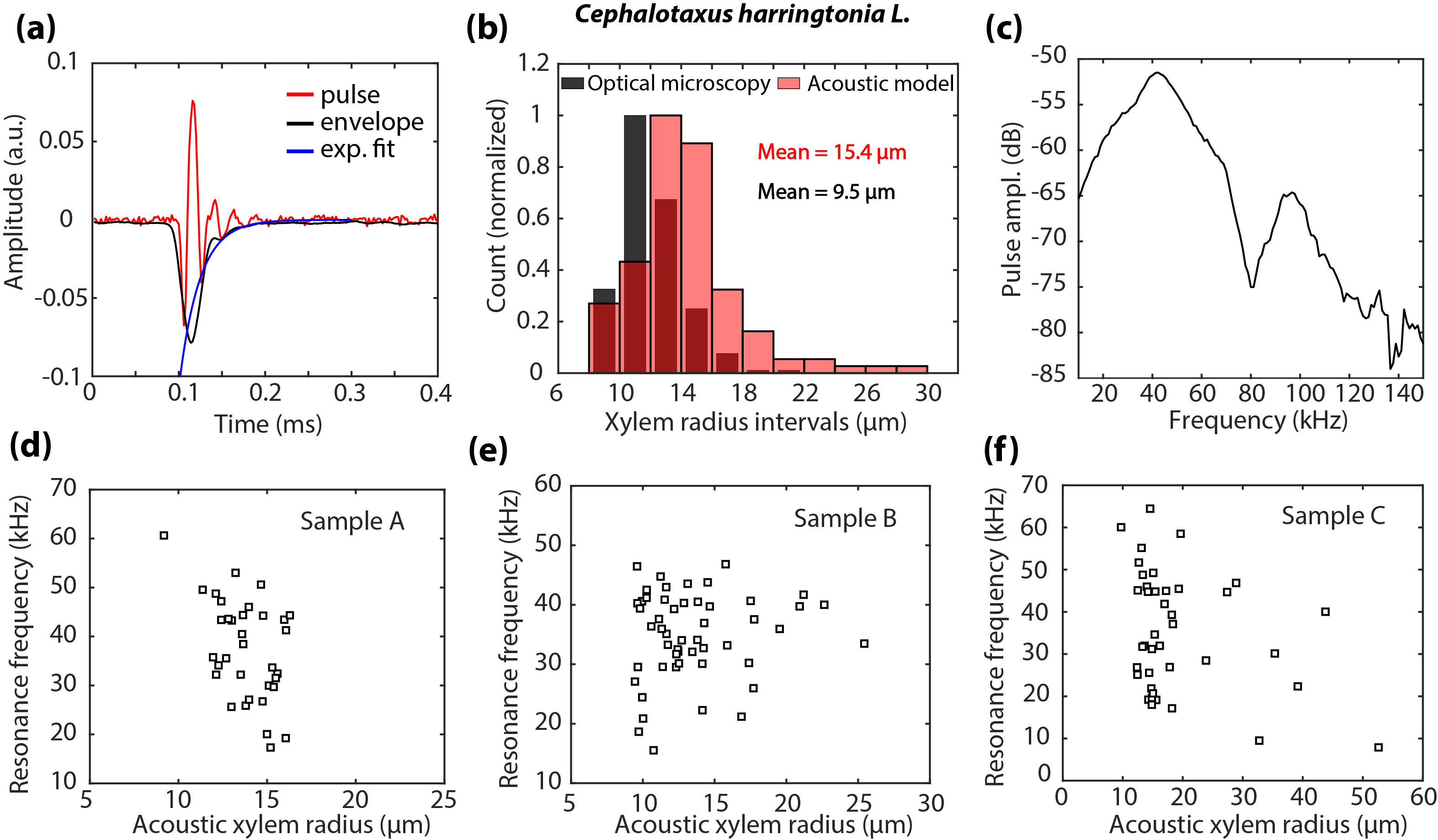


Fig. S4.

**Ultrasound pulse analysis for *Cephalotaxus harringtonia***. **(a)** Zoomed-in time-domain waveform of an example ultrasound pulse from Cephalotaxus stem, recorded axially (**Fig. 1a**). The recording is done with a M500-USB microphone from Pettersson Elektronik AB. Black curves represent the amplitude envelope, and the blue curve represents the exponential fit of the pulse envelope (**Fig. 1c, 1d,** see Materials and Methods). **(b)** Histogram showing the model-extracted xylem radii (in red), and that of the observed xylem radii (in black) obtained via optical microscopy. **(c)** Fourier transform of the example ultrasound pulse shown in (a) showing the characteristic peak frequencies. **(d)-(f)** Model-extracted resonance frequency versus acoustic xylem radius for sound pulses from stem samples A, B and C, respectively. Resonance frequency is obtained from the peak frequency of highest amplitude in the recorded pulses.

**Fig. S5**.


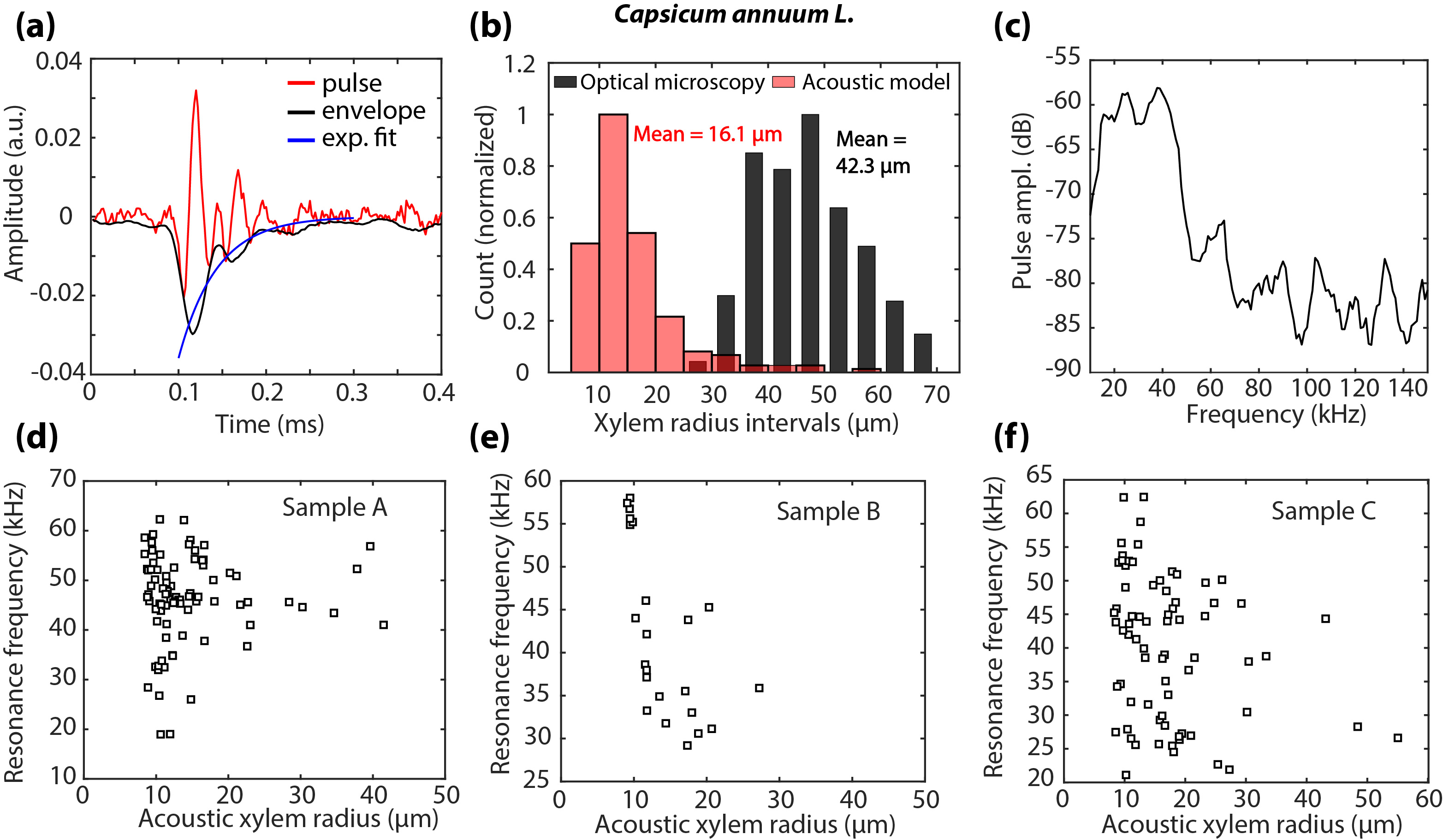


**Ultrasound pulse analysis for *Capsicum annuum***. **(a)** Zoomed-in time-domain waveform of an example ultrasound pulse from Capsicum stem, recorded axially (**Fig. 1a**). The recording is done with a M500-USB microphone from Pettersson Elektronik AB. Black curves represent the amplitude envelope, and the blue curve represents the exponential fit of the pulse envelope (**Fig. 1c, 1d,** see Materials and Methods). **(b)** Histogram showing the model-extracted xylem radii (in red), and that of the observed xylem radii (in black) obtained via optical microscopy. **(c)** Fourier transform of the example ultrasound pulse shown in (a) showing the characteristic peak frequencies. **(d)-(f)** Model-extracted resonance frequency versus acoustic xylem radius for sound pulses from stem samples A, B and C, respectively. Resonance frequency is obtained from the peak frequency of highest amplitude in the recorded pulses.


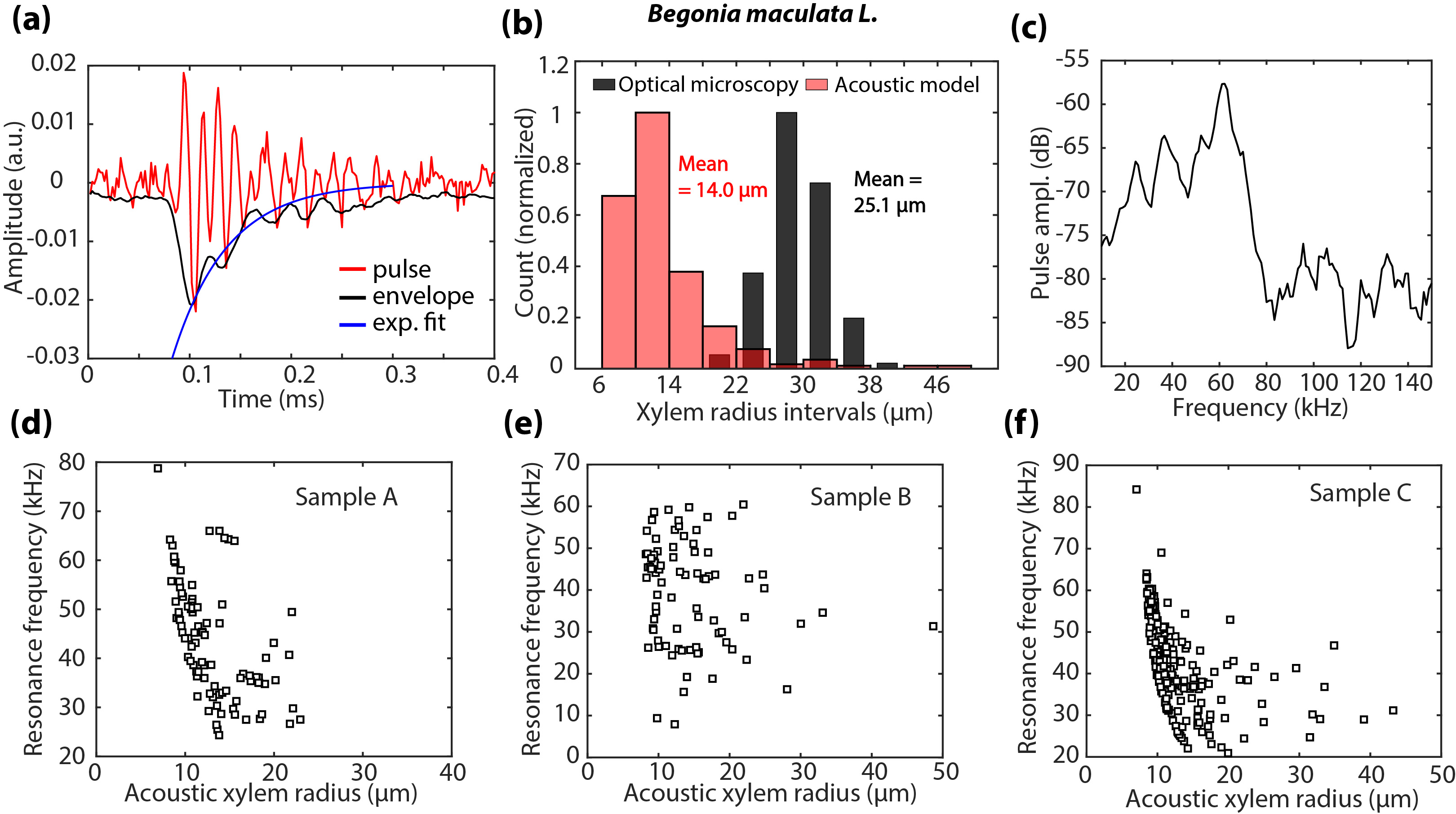


Fig. S6.

**Ultrasound pulse analysis for *Begonia maculata***. **(a)** Zoomed-in time-domain waveform of an example ultrasound pulse from Begonia stem, recorded axially (**Fig. 1a**). The recording is done with a M500-USB microphone from Pettersson Elektronik AB. Black curves represent the amplitude envelope, and the blue curve represents the exponential fit of the pulse envelope (**Fig. 1c, 1d,** see Materials and Methods). **(b)** Histogram showing the model-extracted xylem radii (in red), and that of the observed xylem radii (in black) obtained via optical microscopy. **(c)** Fourier transform of the example ultrasound pulse shown in (a) showing the characteristic peak frequencies. **(d)-(f)** Model-extracted resonance frequency versus acoustic xylem radius for sound pulses from stem samples A, B and C, respectively. Resonance frequency is obtained from the peak frequency of highest amplitude in the recorded pulses.


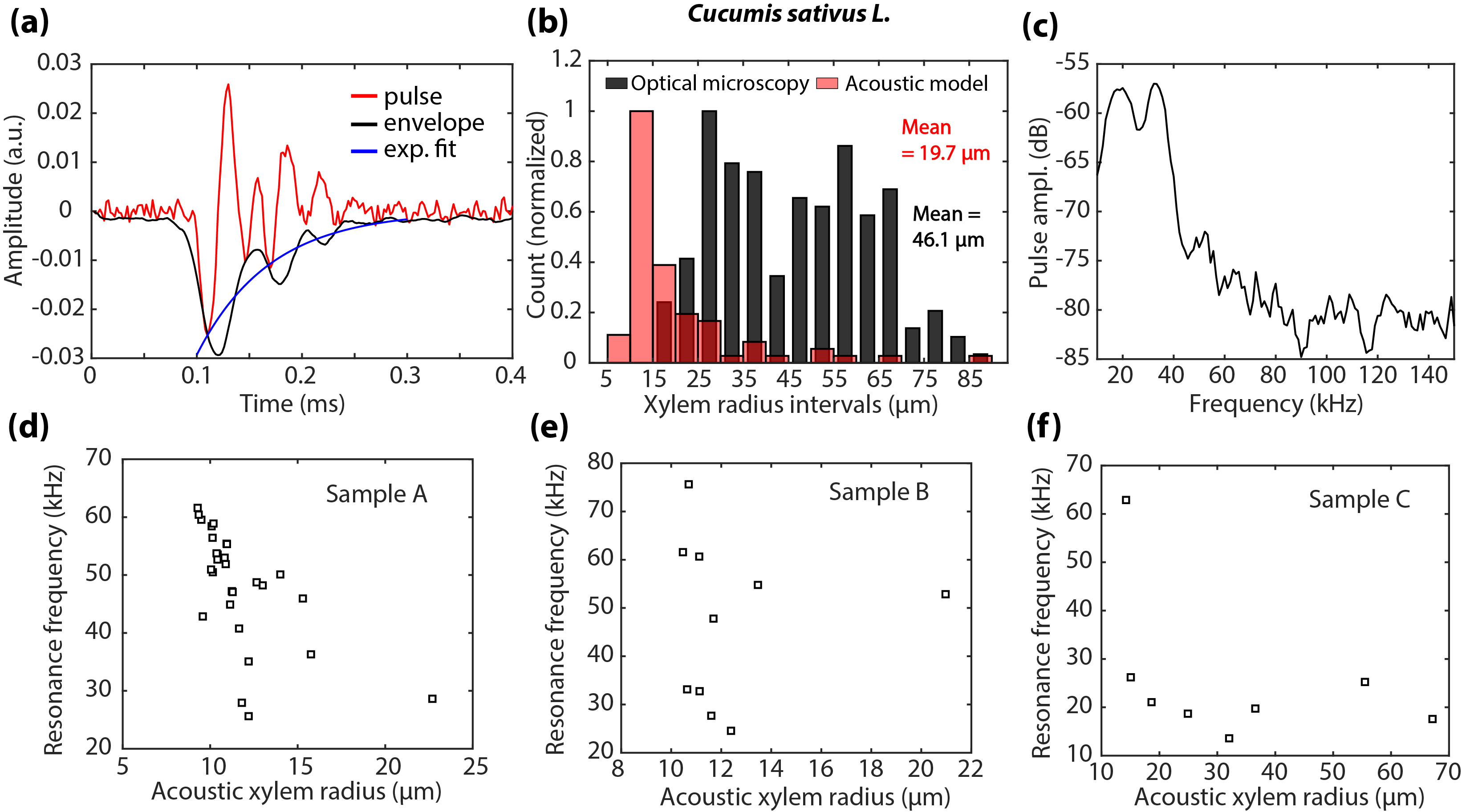


Fig. S7.

**Ultrasound pulse analysis for *Cucumis sativus***. **(a)** Zoomed-in time-domain waveform of an example ultrasound pulse from Cucumis stem, recorded axially (**Fig. 1a**). The recording is done with a M500-USB microphone from Pettersson Elektronik AB. Black curves represent the amplitude envelope, and the blue curve represents the exponential fit of the pulse envelope (**Fig. 1c, 1d,** see Materials and Methods). **(b)** Histogram showing the model-extracted xylem radii (in red), and that of the observed xylem radii (in black) obtained via optical microscopy. **(c)** Fourier transform of the example ultrasound pulse shown in (a) showing the characteristic peak frequencies. **(d)-(f)** Model-extracted resonance frequency versus acoustic xylem radius for sound pulses from stem samples A, B and C, respectively. Resonance frequency is obtained from the peak frequency of highest amplitude in the recorded pulses.


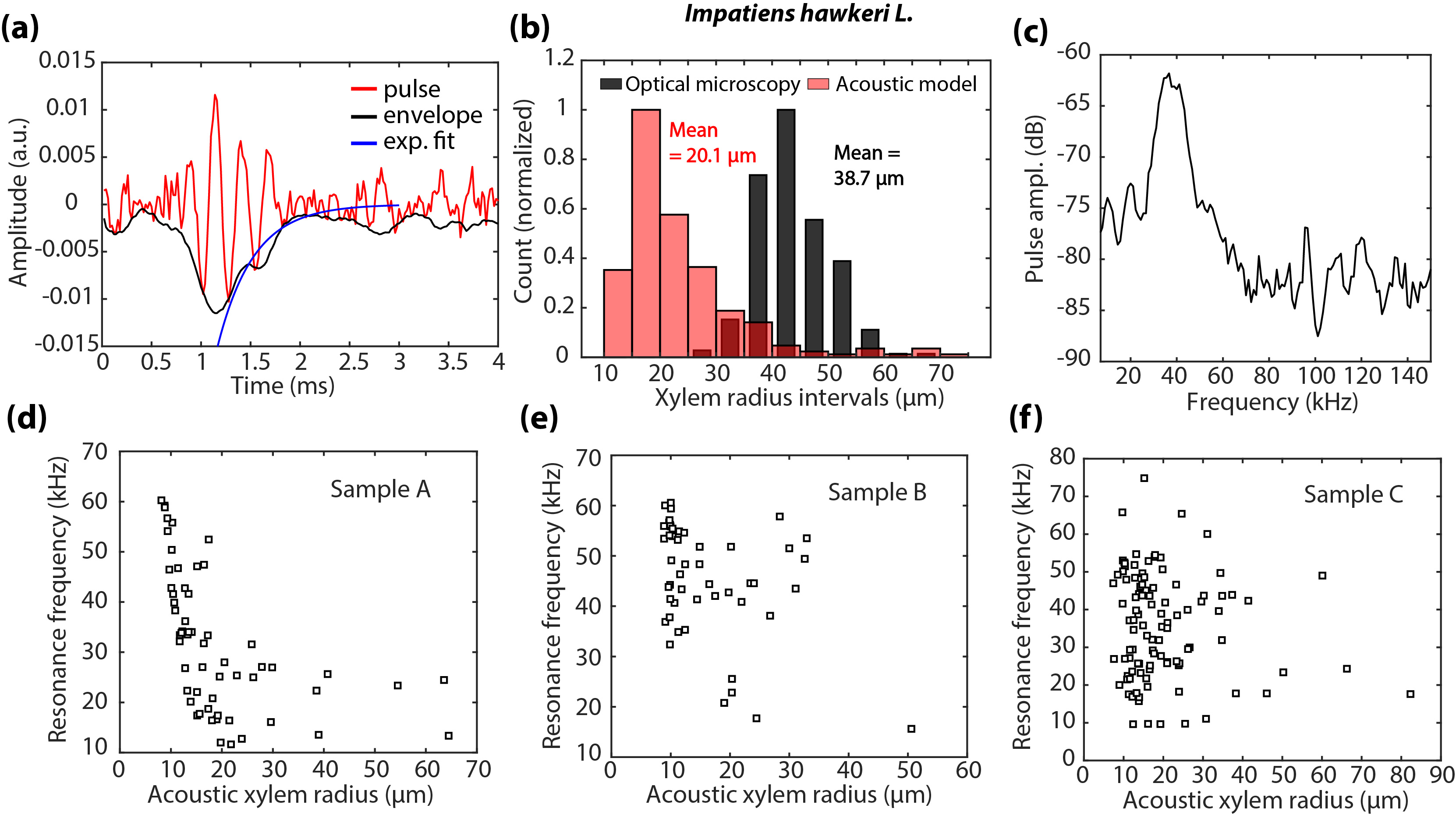


Fig. S8.

**Ultrasound pulse analysis for *Impatiens hawkeri***. **(a)** Zoomed-in time-domain waveform of an example ultrasound pulse from Impatiens stem, recorded axially (**Fig. 1a**). The recording is done with a M500-USB microphone from Pettersson Elektronik AB. Black curves represent the amplitude envelope, and the blue curve represents the exponential fit of the pulse envelope (**Fig. 1c, 1d,** see Materials and Methods). **(b)** Histogram showing the model-extracted xylem radii (in red), and that of the observed xylem radii (in black) obtained via optical microscopy. **(c)** Fourier transform of the example ultrasound pulse shown in (a) showing the characteristic peak frequencies. **(d)-(f)** Model-extracted resonance frequency versus acoustic xylem radius for sound pulses from stem samples A, B and C, respectively. Resonance frequency is obtained from the peak frequency of highest amplitude in the recorded pulses.


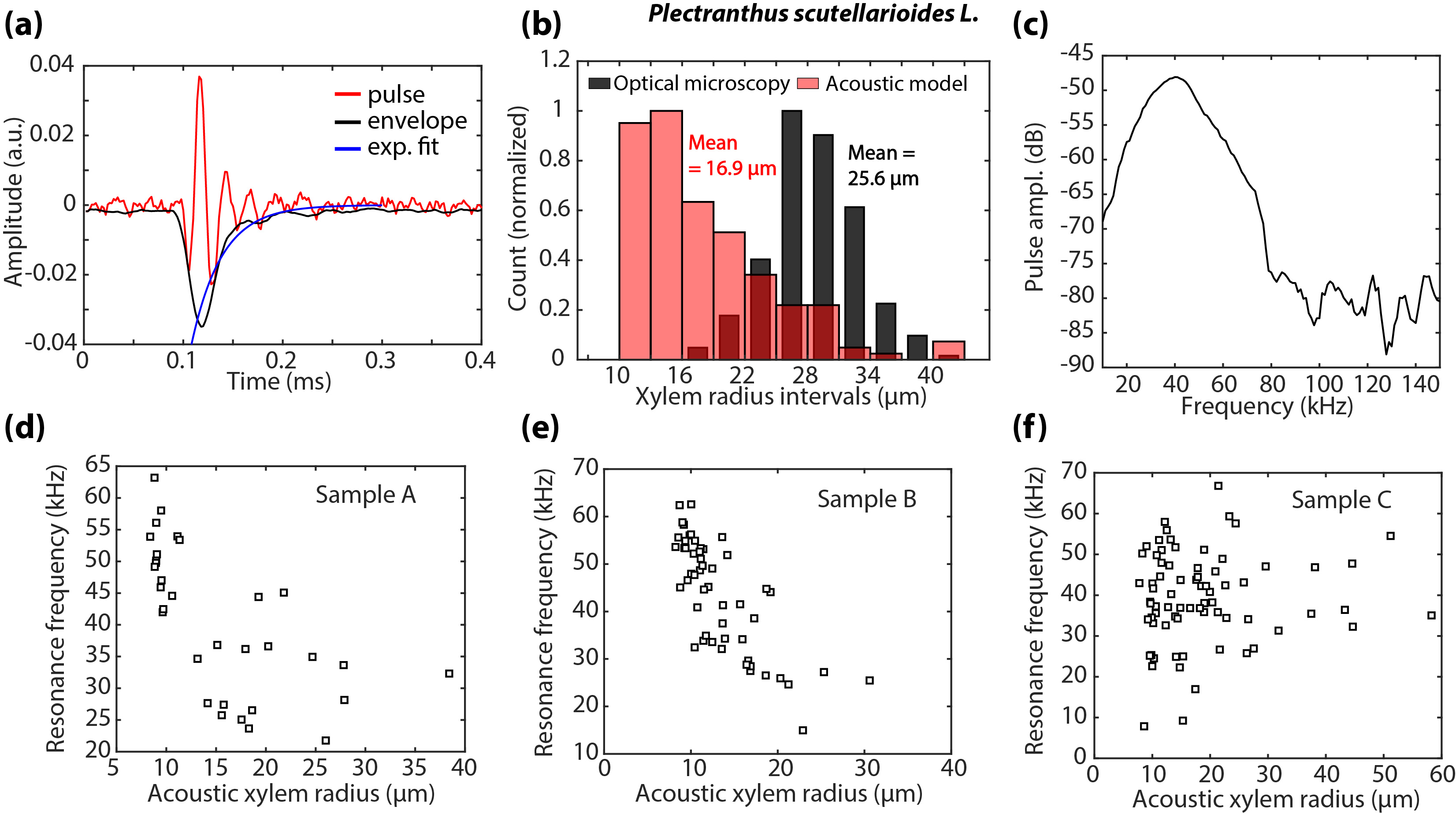


Fig. S9.

**Ultrasound pulse analysis for *Plectranthus scutellarioides***. **(a)** Zoomed-in time-domain waveform of an example ultrasound pulse from Plectranthus stem, recorded axially (**Fig. 1a**). The recording is done with a M500-USB microphone from Pettersson Elektronik AB. Black curves represent the amplitude envelope, and the blue curve represents the exponential fit of the pulse envelope (**Fig. 1c, 1d,** see Materials and Methods). **(b)** Histogram showing the model-extracted xylem radii (in red), and that of the observed xylem radii (in black) obtained via optical microscopy. **(c)** Fourier transform of the example ultrasound pulse shown in (a) showing the characteristic peak frequencies. **(d)-(f)** Model-extracted resonance frequency versus acoustic xylem radius for sound pulses from stem samples A, B and C, respectively. Resonance frequency is obtained from the peak frequency of highest amplitude in the recorded pulses.


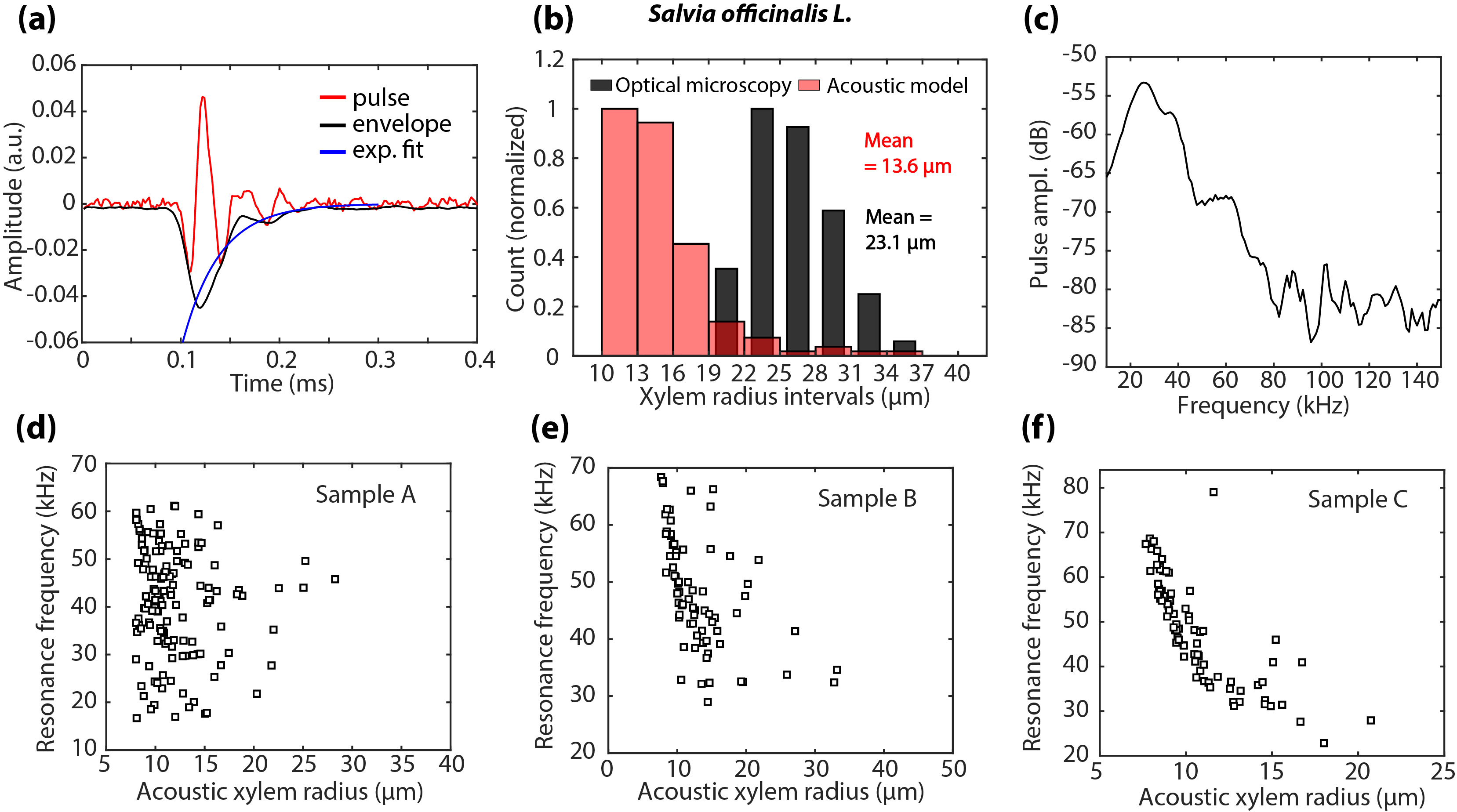


Fig. S10.

**Ultrasound pulse analysis for *Salvia officinalis***. **(a)** Zoomed-in time-domain waveform of an example ultrasound pulse from Salvia stem, recorded axially (**Fig. 1a**). The recording is done with a M500-USB microphone from Pettersson Elektronik AB. Black curves represent the amplitude envelope, and the blue curve represents the exponential fit of the pulse envelope (**Fig. 1c, 1d,** see Materials and Methods). **(b)** Histogram showing the model-extracted xylem radii (in red), and that of the observed xylem radii (in black) obtained via optical microscopy. **(c)** Fourier transform of the example ultrasound pulse shown in (a) showing the characteristic peak frequencies. **(d)-(f)** Model-extracted resonance frequency versus acoustic xylem radius for sound pulses from stem samples A, B and C, respectively. Resonance frequency is obtained from the peak frequency of highest amplitude in the recorded pulses.


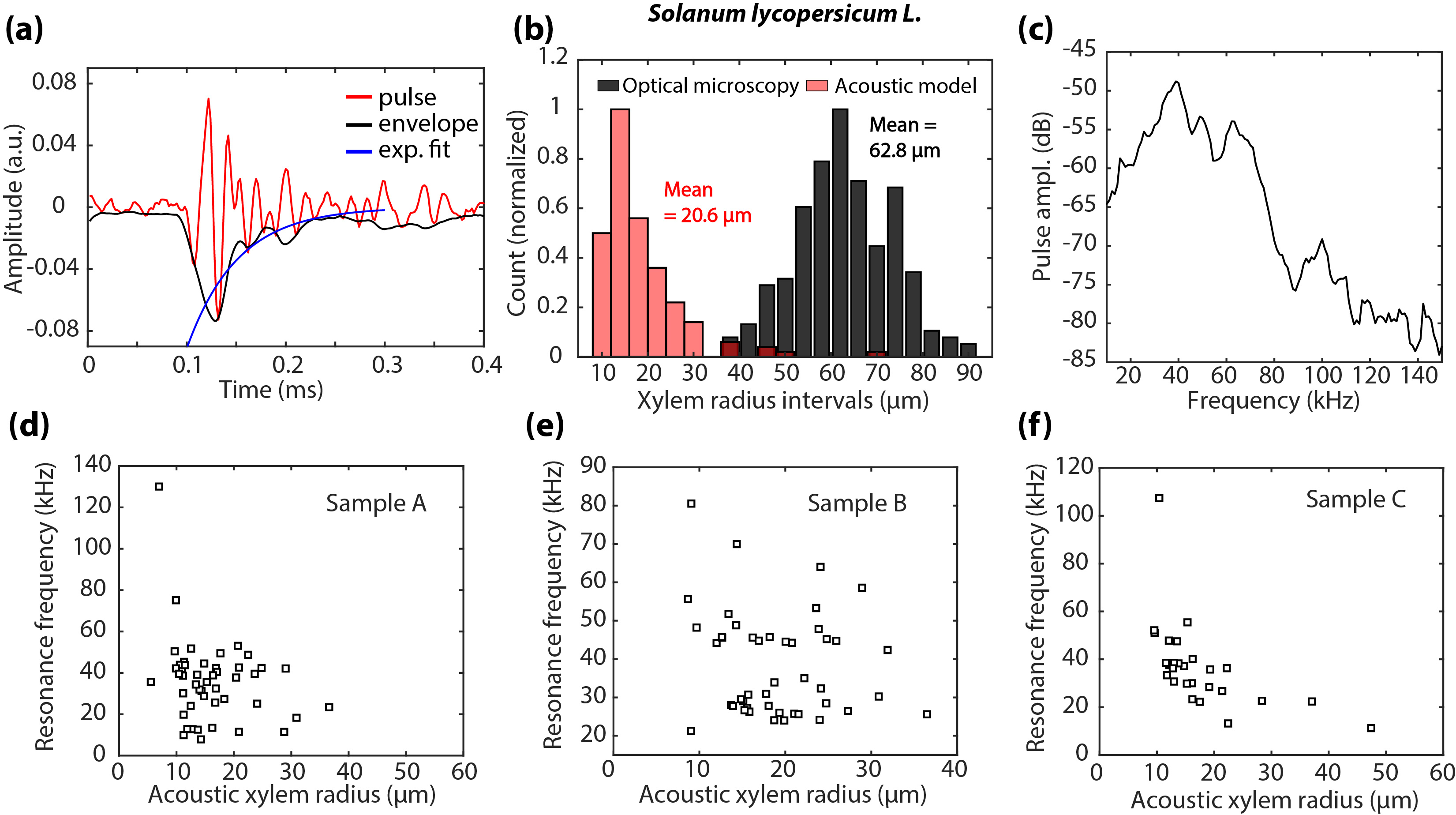


Fig. S11.

**Ultrasound pulse analysis for *Solanum lycopersicum***. **(a)** Zoomed-in time-domain waveform of an example ultrasound pulse from Solanum stem, recorded axially (**Fig. 1a**). The recording is done with a M500-USB microphone from Pettersson Elektronik AB. Black curves represent the amplitude envelope, and the blue curve represents the exponential fit of the pulse envelope (**Fig. 1c, 1d,** see Materials and Methods). **(b)** Histogram showing the model-extracted xylem radii (in red), and that of the observed xylem radii (in black) obtained via optical microscopy. **(c)** Fourier transform of the example ultrasound pulse shown in (a) showing the characteristic peak frequencies. **(d)-(f)** Model-extracted resonance frequency versus acoustic xylem radius for sound pulses from stem samples A, B and C, respectively. Resonance frequency is obtained from the peak frequency of highest amplitude in the recorded pulses.


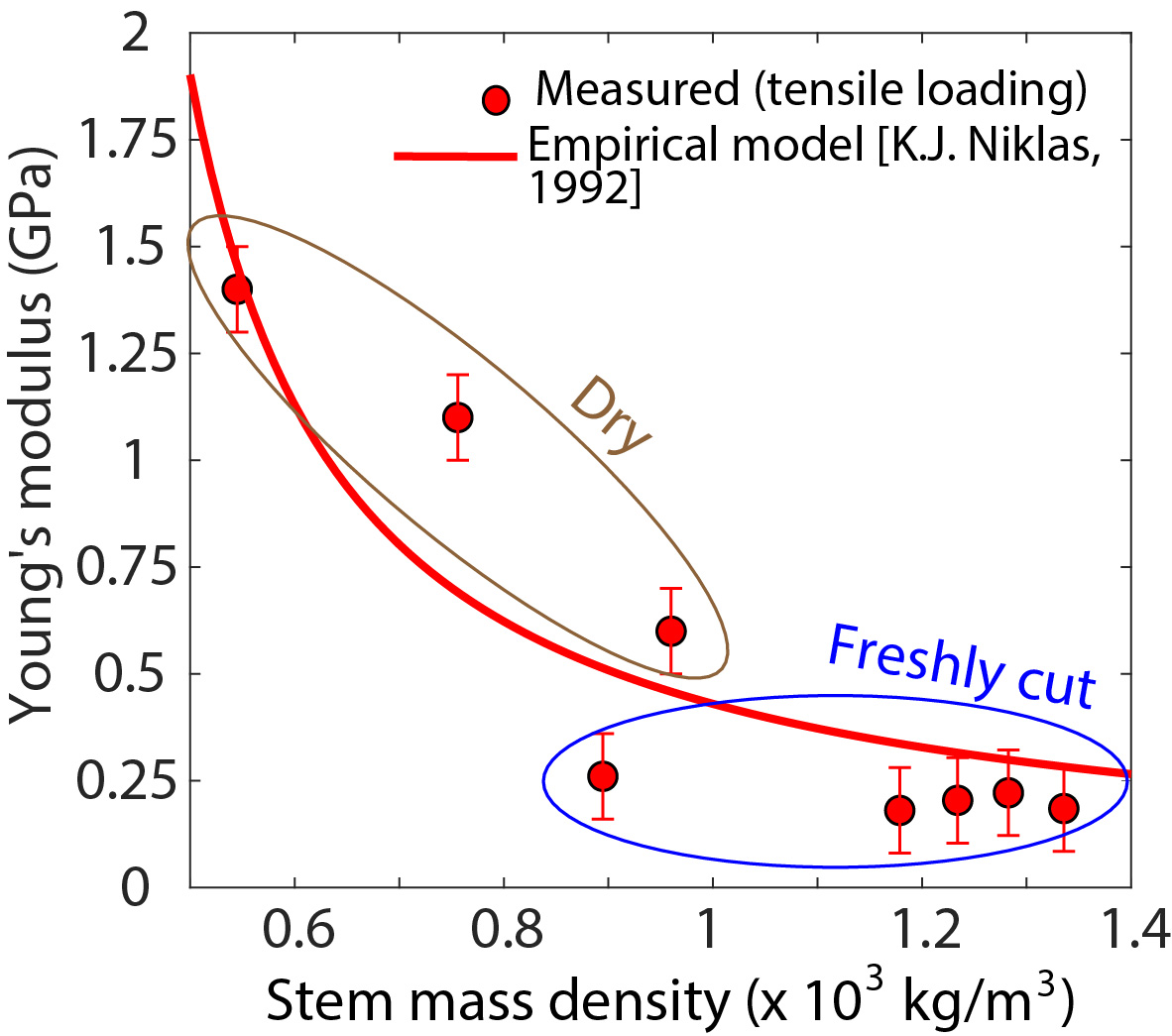


Fig. S12.

**Young’s moduli of *H. quercifolia* stem segments.** Extracted Young’s modulus versus mass density for freshly cut and dried stem segments, extracted from longitudinal stress-strain measurements (solid circles) with indicated error bars (± 0.1 GPa). The red curve represents a fit based on the empirical model [24] of Young’s moduli as a function of relative water-content in stems.


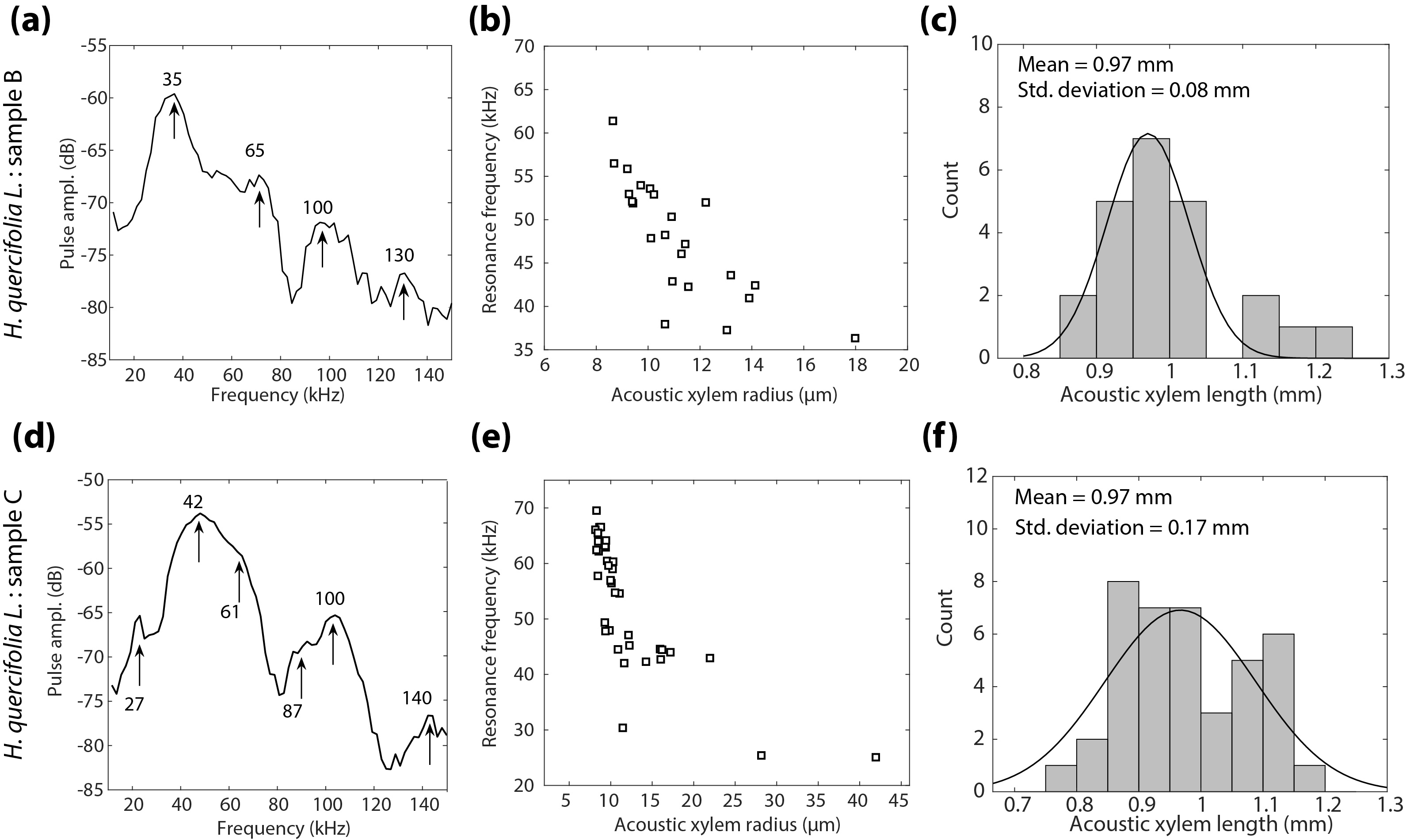


Fig. S13.

**Ultrasound frequency spectra, resonance frequencies and acoustic length of xylem vessel element in *H. quercifolia*.** **(a)** Observed characteristic peak-frequencies in the example Fourier transform of the ultrasound pulses recorded axially in Hydrangea stem sample B. The black curve represents the spectrum of a representative pulse with the indicated timestamp of the recording. **(b)** Model-extracted resonance frequency versus acoustic xylem radius for sound pulses from stem sample B. Resonance frequency is obtained from the peak frequency of highest amplitude in the recorded pulses. **(c)** Histogram showing the extracted xylem vessel element lengths in stem sample B via the acoustic model. The black curve represents a unimodal Gaussian fit. **(d)-(f)** Stem sample C: Observed characteristic peak-frequencies in the example Fourier transform of the ultrasound pulses recorded axially; model-extracted resonance frequency versus acoustic xylem radius; histogram of the model-extracted xylem vessel element lengths.


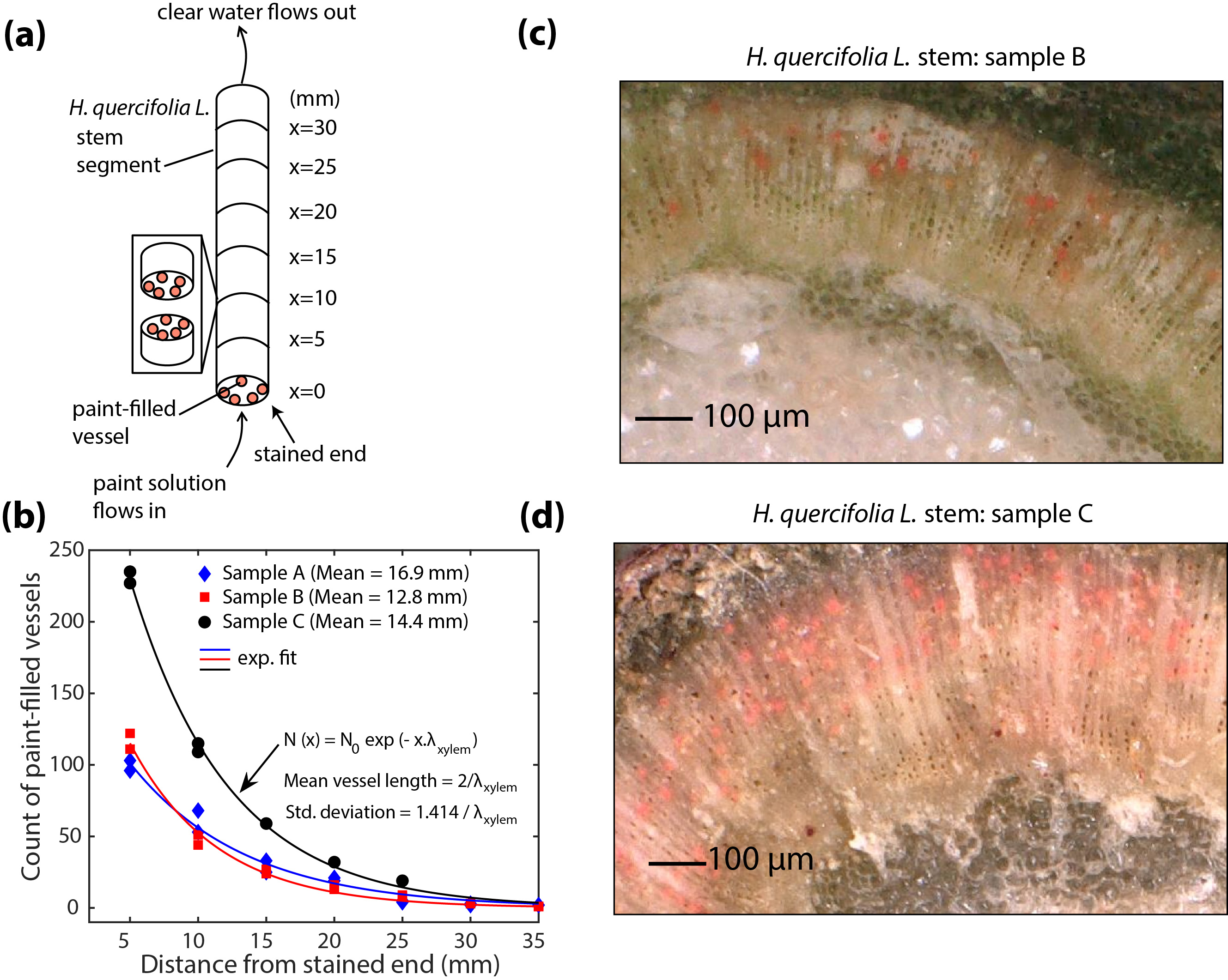


Fig. S14.

**Latex paint infusion, vessel counting and optical microscopy in *H. quercifolia*.** **(a)** Schematic illustration of the latex paint-infusion method. The paint solution is sucked in from the bottom end. Water passes through the perforation plates and border pits on the walls of xylem vessels and emerges out from the top end. The paint molecules fill up all the vessel elements inside a single xylem vessel and gets blocked by its fused ends. The infused stem sample is sectioned at 5 mm intervals along the length from the paint-infiltration end. **(b)** Number of paint-filled vessels as a function of position *x* along the stem (symbols). The solid curves represent the exponential fits. The characteristic length *λ*_xylem_ is the reciprocal of the most probable vessel length. **(c), (d)** Optical micrographs (200X) of the transverse cross-sections of the of Hydrangea stem samples B and C respectively, cut at 5 mm distance from the paint-infusion end. The paint-filled vessels can be seen in fluorescent red colour.


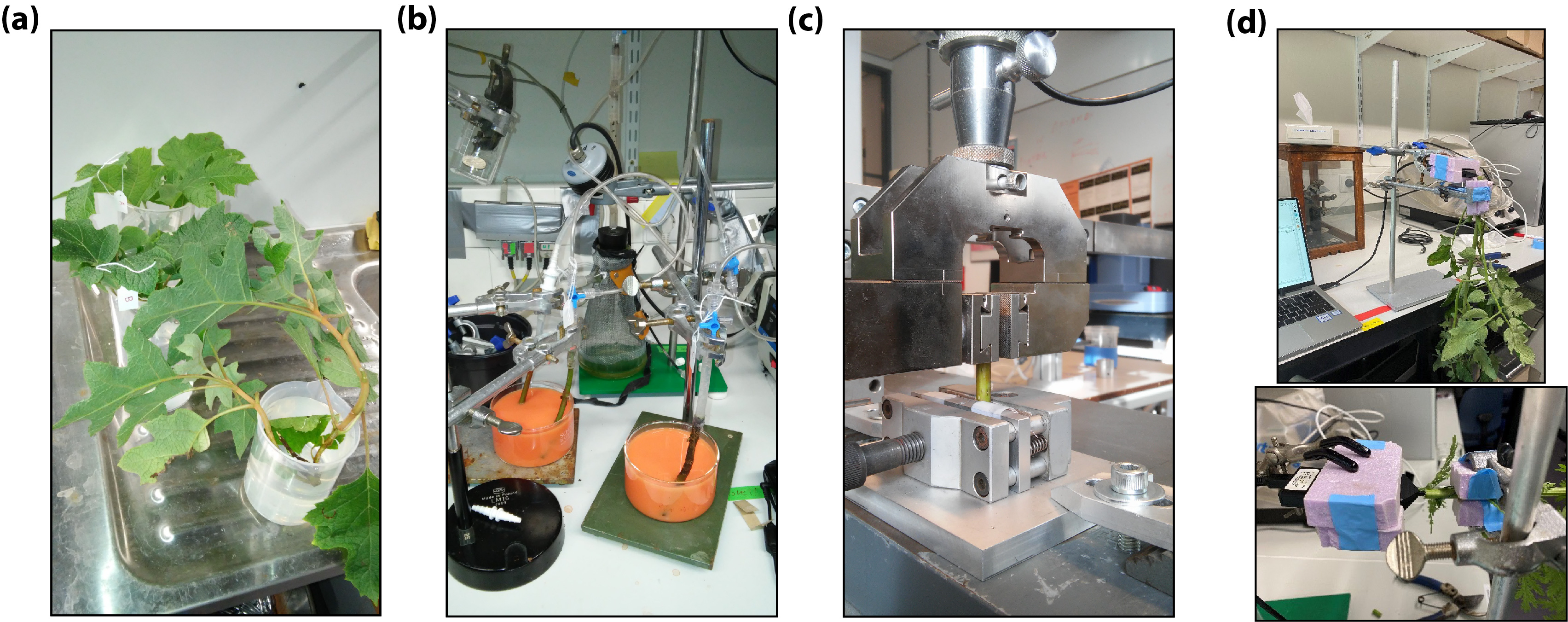


Fig. S15.

**Photographs from experiments.** **(a)** A shoot of Hydrangea placed in water immediately after cutting it from the main plant under water. The shoot is then used for ultrasound recording and a cut-out stem segment from the same is used for latex paint-staining experiment. **(b)** Set-up of the latex paint-staining apparatus with the stem segments from samples A, B, and C mounted vertically over the paint vessels. **(c)** Set-up of the Zwick/Roell uniaxial tensile testing machine with the stem segment mounted vertically between the clamps. **(d)** Set-up showing the process of ultrasound recording from a cut Solanum shoot using the M500-USB microphone fastened on a clamp stand surrouned by vibration isolation foam.
